# Sea ice variability and maritime activity around Svalbard in the period 2012–2019

**DOI:** 10.1038/s41598-020-74064-2

**Published:** 2020-10-12

**Authors:** Alexandra N. Stocker, Angelika H. H. Renner, Maaike Knol-Kauffman

**Affiliations:** 1 Department of Geography, Umeå University, 901 87 Umeå, Sweden; 2 University Center of the Westfjords, Isafjordur, Iceland; 3 Department of Oceanography and Climate, Institute of Marine Research, Fram Centre, 9296 Tromsø, Norway; 4 Norwegian College of Fishery Sciences, University of Tromsø - The Arctic University of Norway, 9037 Tromsø, Norway

**Keywords:** Cryospheric science, Environmental impact, Sustainability

## Abstract

Climate change is strongly impacting the Arctic environment, leading to rapid sea ice loss. In some sectors, the retreating ice edge is perceived as an opportunity to expand and develop economic activities. Previous studies show this development in the Canadian and Russian Arctic. This paper examines mobility patterns of cruise ships and fishing vessels around Svalbard, a major hotspot of maritime activity and retreating sea ice cover, in relation to sea ice variability between August 2012 and September 2019. The results show a slight overall increase in fisheries and cruise activity, as well as remarkable trends of stretching operational seasons and expanding navigational areas in these sectors. Overall increasing activity and changing mobility patterns provoke a discussion about the implications for safe navigation and sustainable management, thus raising issues of high pan-Arctic relevance.

## Introduction

The polar regions are the most climate-sensitive areas in the world, and temperatures in the Arctic are rising more than twice as fast as the global average^1,2^. This increase in temperatures results in large changes in sea ice conditions, with both thickness and extent following negative trends^1,3,4^. Sea ice is a large impediment for the development of maritime activities in the High Arctic and limits the activity to seasonal operations in most areas. Therefore, the decline of sea ice is opening new opportunities for maritime activities to expand^5–7^. The fastest developing sectors are shipping, tourism and recreational craft^1^, resulting in an increase in the number of ships in the Arctic by 25% between 2013 and 2019^8^. This increase in vessels entails high risks and raises strong concerns about environmental impacts and safety issues as it puts pressure on supporting infrastructures and regulations^5^.

Safety concerns regarding high latitude cruise tourism and fishing operations are progressively put on the political agenda, nationally and internationally. In 2017, the International Maritime Organization (IMO) implemented the Polar Code, which sets mandatory requirements for design, construction, equipment, operational, training, search and rescue and environmental protection matters for all vessels sailing in polar waters^9^. More recently on a national level, the Norwegian Government in its latest Svalbard strategy emphasized the need to asses preparedness and safety challenges arising from increased shipping activity around the archipelago of Svalbard^10^. From a pan-Arctic perspective, there is a high level of maritime activity in Svalbard waters, together with the rest of the Barents Sea, and notably the Kara Sea^11,12^. At the same time, the region experiences the most drastic environmental changes in the Arctic with the northward retreat of the ice edge^13^, the delay of winter freeze-up^14^, and the air temperatures rising faster than global or even Arctic average^15^. Svalbard, therefore, serves as a relevant case study for the development of maritime activities and environmental change, and can provide important lessons applicable to other Arctic regions.

**Figure 1:**
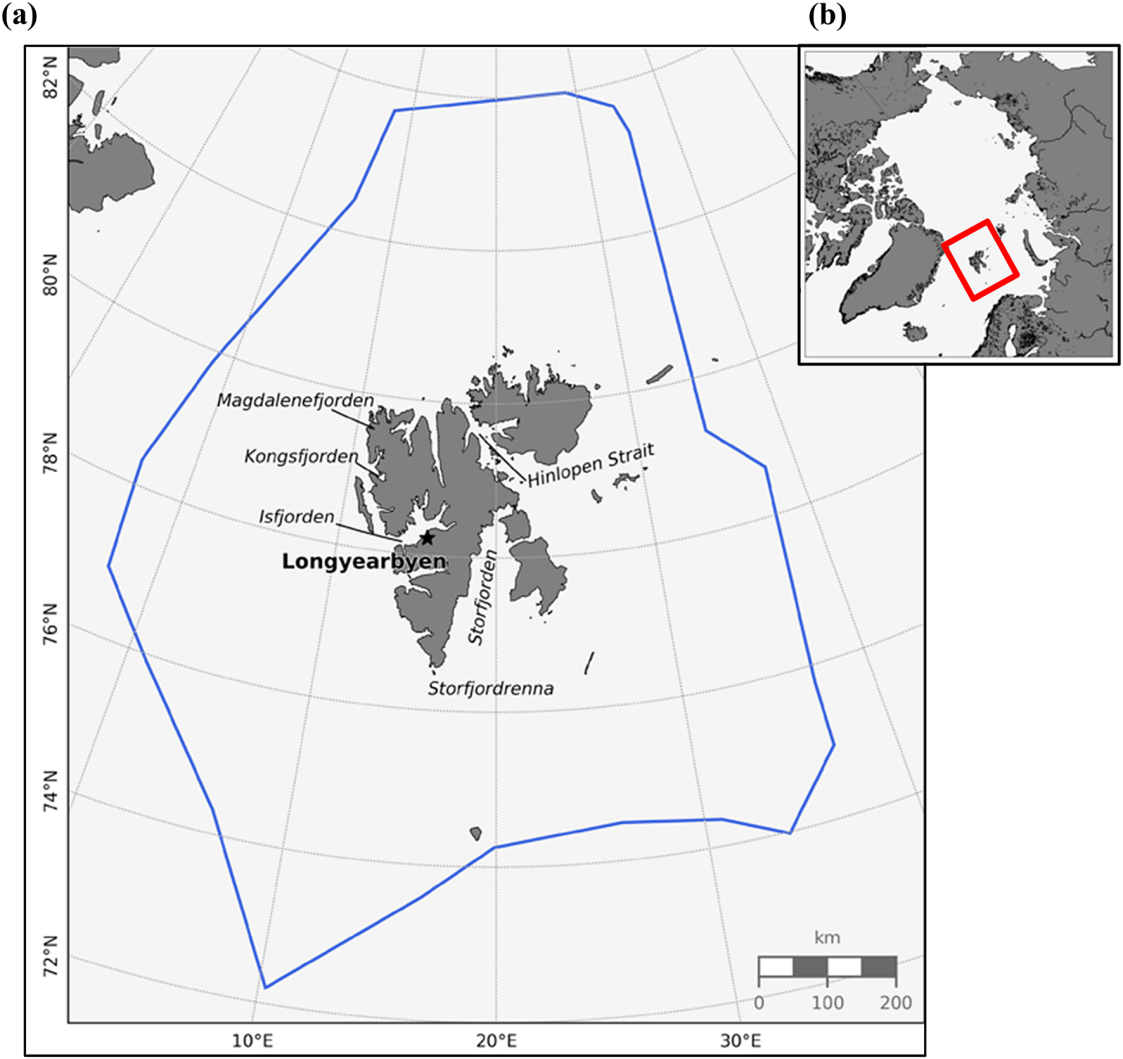
(**a**) Map of Svalbard with main administrative settlement Longyearbyen and relevant geographical areas where navigation takes place. The blue line delimits the boundaries of the SFPZ. (**b**) Location of the archipelago in the Arctic. Figures generated with python 3.7.x (https://www.python.org/).

While maritime activity around Svalbard has increased in the past decade, to date the actual developments have not been mapped in a comprehensive way. The objective of this work is to visualize and discuss the relationship between marine economic activities (cruise tourism and fisheries), and sea ice variability around Svalbard between 2012 and 2019, in order to understand the potential implications for safety and sustainable management. The study area is delimited by the Svalbard Fisheries Protection Zone (SFPZ), which reaches out to 200 nm from the coastlines and is roughly located between 72° N and 84° N, and 3° W and 38° E (Fig. 1). The analysis is based on two datasets: (1) sea ice concentration and (2) Automatic Identification System (AIS) data. These datasets are complemented with seven semi-structured interviews with industry and public policy representatives as well as sea ice service providers, which enabled us to reflect on how sea ice conditions influence decision-making in the fisheries and cruise industries.

The cruise tourism sector is an important pillar for Svalbard’s economy^16^ and has rapidly increased over the past decade, where the total numbers of cruise passengers rose by 73% between 2008 and 2018^17^ (Table 2). In parallel, retreating sea ice over the summer months has resulted in a prolongation of the season^18^. Most studies that analyzed the relationship between sea ice and cruise tourism focus on the Canadian Arctic, where the number of these vessels has tripled since 1990^19^. Fisheries are the dominant fleet in the Barents Sea and Svalbard area in terms of the number of vessels and their year-round operations^11,20^. This region is facing large climate-driven changes in fish community structure^21^. The most common and commercially important species in the boreal community are cod and haddock^22^, and their northward migration^23^ results in more fishing activity at higher latitudes^20^. This paper contributes new contextual knowledge regarding the number and mobility patterns of these sectors in the European Arctic around Svalbard.

The remaining of this paper is structured as follows. The results section analyzes the number of vessels sailing around Svalbard and the seasonal and geographical stretching of operations between August 2012 and September 2019. The discussion elaborates on how changing mobility patterns of cruise ships and fishing vessels raise concerns for safety and sustainability in Svalbard waters. The conclusion reflects on our methodological approach and outlines complementary ways to achieve a comprehensive understanding of maritime change and its potential implications in Arctic regions.

## Results

### Total maritime activity around Svalbard

**Figure 2:**
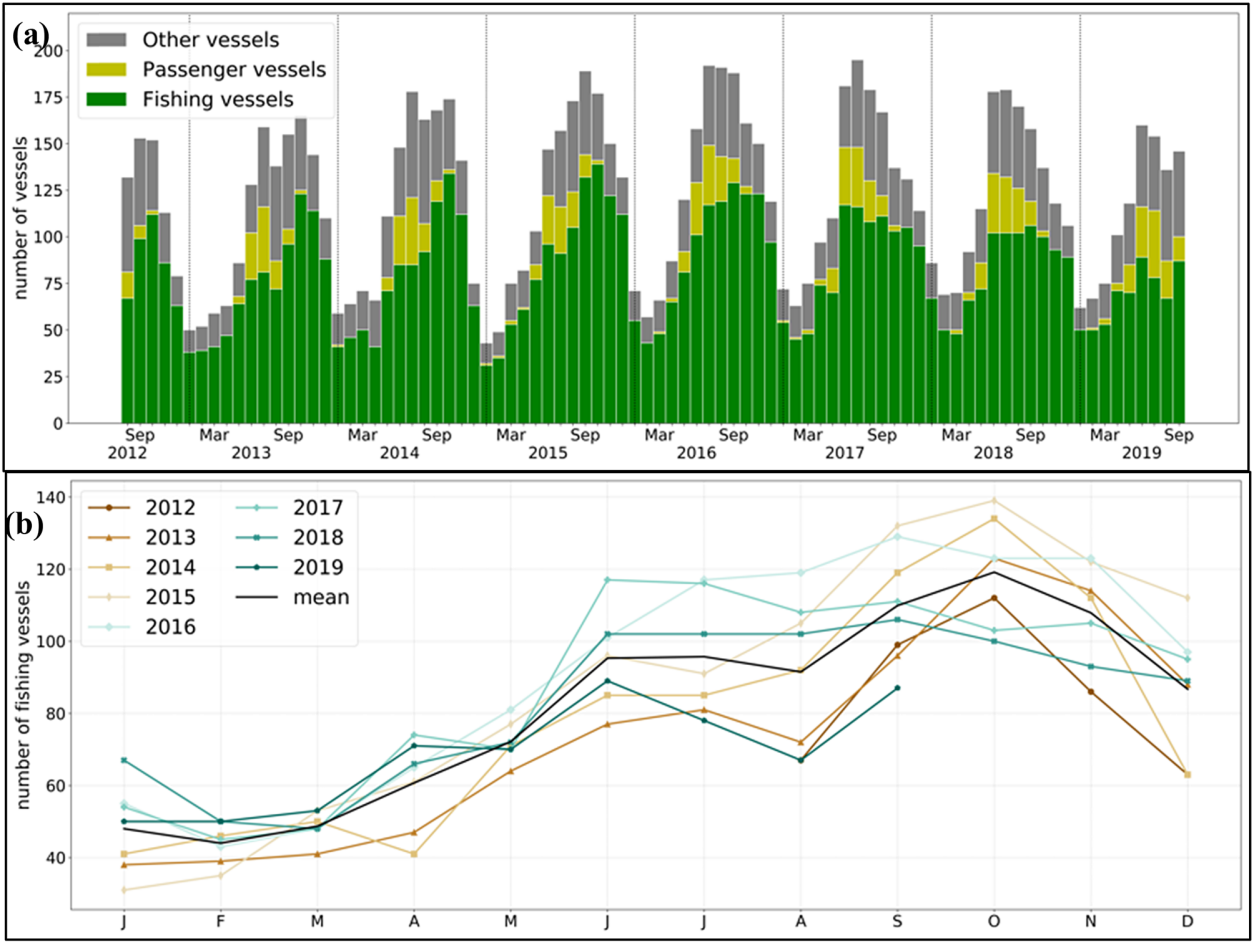
(**a**) Number of vessels in SFPZ from August 2012 until September 2019. Green: fishing vessels, yellow: passenger vessels (oversea and expedition cruise ships), grey: other categories (research, container ships, bulk carrier, etc.…). (**b**) The number of fishing vessels per month over the study period.

Overall, maritime activity is increasing in the Arctic. To determine the changes in mobility patterns around Svalbard, we analyzed the number of vessels sailing in the SFPZ using AIS data. Independently of the vessels’ trajectories, each unique vessel was counted once per month, and the time series between 2012 and 2019 shows that more vessels were present in the later years (Fig. 2). However, the total annual number of vessels shows consistency over the study period (Table 1). Therefore, the same vessels are operating for longer time periods in the SFPZ, increasing monthly activity. The highest activity observed is between July and October, with an average of 165 vessels per month. July 2017 was the busiest month in the study period with 195 vessels, which is a nearly 23% increase from July 2013 (159 vessels). The lowest activity took place between January and March in all years, where an average of 66 vessels sailed per month. Overall, throughout the study period, fishing vessels have slightly increased in number (Fig. 2), while the annual number of passenger vessels has remained fairly stable (Table 1).

**Table 1:** The total number of unique vessels that sailed in the SFPZ per year.

	2012*	2013	2014	2015	2016	2017	2018	2019**
Number of passenger vessels	14	45	43	33	44	42	40	42
Number of fishing vessels	151	186	204	223	222	213	208	171
Number of other vessels	79	107	114	106	95	105	102	102
Total	244	338	361	362	361	360	350	315

*August–December.

** January–September.

Fishing vessels accounted for more than half of all vessels operating in the SFPZ in each month, making it the dominant fleet in this region. The number of fishing vessels has increased since 2014 (Table 1), and while this increase in the SFPZ can likely be explained by the northward migration of important commercial boreal species, such as cod and haddock^21,23^, this does not reflect official catch reports for the Northeast Arctic region, showing relatively stable catches of these species over the study period^24,25^. Another potential explanation for the increase in the number of fishing vessels is that more vessels have become equipped with AIS transponders. The law gradually extended for fishing vessels registered in the European Union and European Economic Agreement area. Since May 2012, it is mandatory for fishing vessels of 24 m and larger to carry AIS transponders; for vessels between 18–24 m and 15–18 m, this is the case from 31 May 2013 and 31 May 2014, respectively^26^. As a result of these European regulations on AIS, the Northeast Atlantic region holds the highest number of fishing vessels above 24 m that send AIS signals^27^, and the majority of vessels under 12 m operate along the shores of continental Europe. Therefore, fishing activity in the SFPZ can be considered to be well represented by AIS data, and we expect that the AIS data does not deviate significantly from the actual growth up to 2014, nor through the rest of the study period. AIS transmission limitations still need to be acknowledged and are explained in the methods section.

Passenger vessels are classified in two main categories: oversea cruise ships and expedition cruise vessels. The first category includes larger vessels that only sail to Longyearbyen (Fig. 1a). The second category includes day-trip cruises in Isfjorden (Fig. 1a) and some pleasure craft that are equipped with AIS transponders. Activity in these two categories has developed differently over the study period. Oversea cruise ships have decreased in number while their passenger capacity increased. In 2018, the “*MSC Meraviglia*'' brought 6′000 people to Svalbard, making it the biggest passenger vessel to operate in the European Arctic. The number of oversea cruise ships visiting Svalbard was higher prior to 2015. Between 1996 and 2014, around 21–35 oversea cruise vessels visited Svalbard each year, whereas from 2015 to 2018 on average 15 vessels visited the archipelago annually^28^.

Svalbard is a popular destination and to a large extent oversea cruise tourism explains the rise of tourists visiting the archipelago (Table 2). Expedition cruise vessels, however, have increased in numbers (Table 2), but followed a trend towards smaller vessels^28^. This explains why the total number of passenger vessels has remained stable over the study period (Table 1). In 2015, both categories of passenger vessels decreased in number, likely due to the expansion of the ban of heavy fuel oils (HFO) that year. This ban was first introduced in the nature reserves in the east and in the national parks in the west of the archipelago in 2007 and 2009, respectively^29^. Today, this ban applies in the majority of territorial waters in national parks and nature reserves around the archipelago^30^. The difference in the total number of passenger vessels between Tables 1 and 2 is due to the fact that our AIS data exclude smaller vessels which accounts for a majority of pleasure craft, which are becoming more popular throughout the Arctic^1^ and are all recorded by the Governor of Svalbard, showing an increase of 42% between 2008 and 2018 (Table 2).

**Table 2:** Increase in the number of cruise passengers visiting the archipelago of Svalbard^17^.

	Number of vessels		Total number of passengers	
	2008	2018	2008	2018
Oversea cruise ships	28	15	28,697	45,900
Expedition cruise vessels	24	59	10,040	21,000

### Stretching of the season

The data show a prolongation of the operational season for both cruise tourism and fishery activity around Svalbard. The main operational season for passenger vessels in the SFPZ was between June and August. However, expedition cruise vessels started to operate earlier in the spring as of 2015 (Fig. 2a). Taking the spring season of 2013 and 2019, the number of vessels went from zero to four in April and four to 15 in May (Fig. 2a). Fisheries were most active in the autumn months in the first part of our study period, but extended their season particularly noticeably into early summer (June/July) and early winter (December; Fig. 2b).

**Figure 3:**
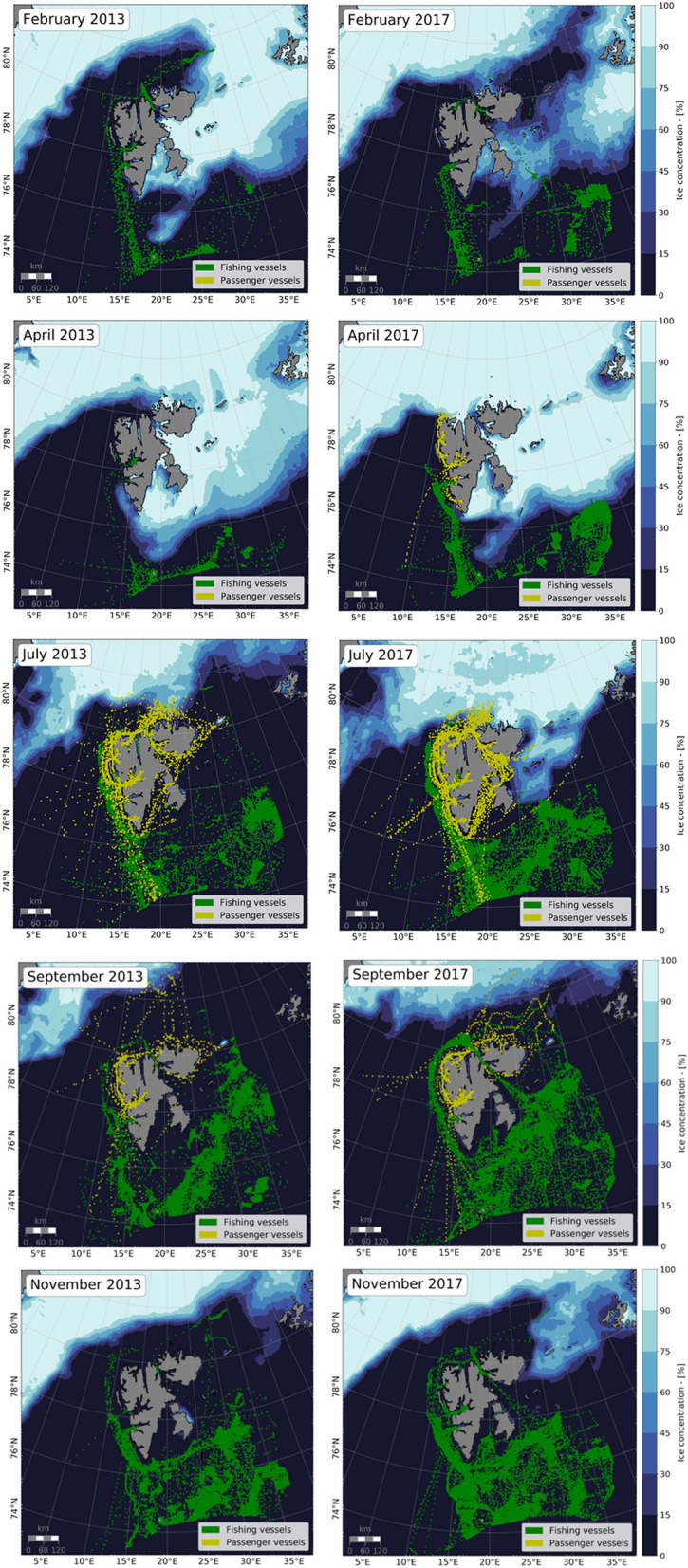
Average sea ice concentration, and fishing (green) and passenger (yellow) vessel positions in February, April, July, September and November of 2013 (left) and 2017(right). Figures generated with python 3.7.x (https://www.python.org/).

To enable a deeper understanding of the relationship between sea ice variability and cruise tourism and fishing activities, maps for all months in the study period were produced and investigated, which are available in the Supplementary material online. Here, we show examples from 2013 and 2017 to visualize seasonal mobility patterns (Fig. 3). These years were selected, because 2013 is the first year with complete data both for AIS and sea ice concentration, and 2017 has a particular large sea ice cover until mid-summer. Two points are important to keep in mind when reading the maps. First, the sea ice cover can change rapidly over a month’s period and the activities most likely take place in open waters or very low sea ice cover. Secondly, previous ice conditions are an important basis for long-term itinerary planning in the cruise industry. Therefore, ice conditions of summer 2016 (see Supplementary Fig. S8; Fig. S9 online) should be taken into account as well when analyzing mobility patterns and number of expedition cruise vessels in summer 2017.

This expansion of seasonal expedition cruising is also reflected in the maps (Fig. 3). In April 2017, there were three vessels sailing along the west coast up to the ice edge near 80°N (Fig. 3), which is considerable activity for this region. The stretching over the spring shoulder season could be explained by the long daylight period (the sun remains above the horizon between April and August), the larger sea ice cover, and the higher chances of wildlife observations^31^. These Arctic features make this season attractive for expedition cruise tourism. Oversea cruise ships have not expanded their operational season, which lasts from June through August. An industry representative argued that these vessels avoid sailing when ice can still be present in Isfjorden, while heading for Longyearbyen (Fig. 1).

For fisheries in the SFPZ, shrimp and cod are the most important species, and fishing opportunities are determined partly by the size and distribution of the stock, and the sea ice extent^20^. A fishery representative explained that the best season for shrimp is between late winter and early summer, while cod fisheries are the richest and most accessible during the autumn months. More intense shrimp fishery could thus explain the increase in activity to early summer (from September early in the study period to June in the later years), whereas less sea ice and better accessibility promoted more cod fishing activity into December (Fig. 2b). For example, in July 2013, 81 fishing vessels sailed in the SFPZ; this number increased by 43% in July 2017 (116 vessels, Fig. 2b), which is also visible in the maps (Fig. 3). This seasonal stretching over the summer months might have resulted in the regression of the autumn activity peak observed in the earlier years (Fig. 2a). The late winter months have not witnessed a clear change throughout the study period, where fishing activity between January and March remained fairly low, as illustrated in the February months in Fig. 3. This low activity could be explained by various reasons such as polar nights, significant sea ice cover and low ecological activity.

### Change in the sailing areas

**Figure 4:**
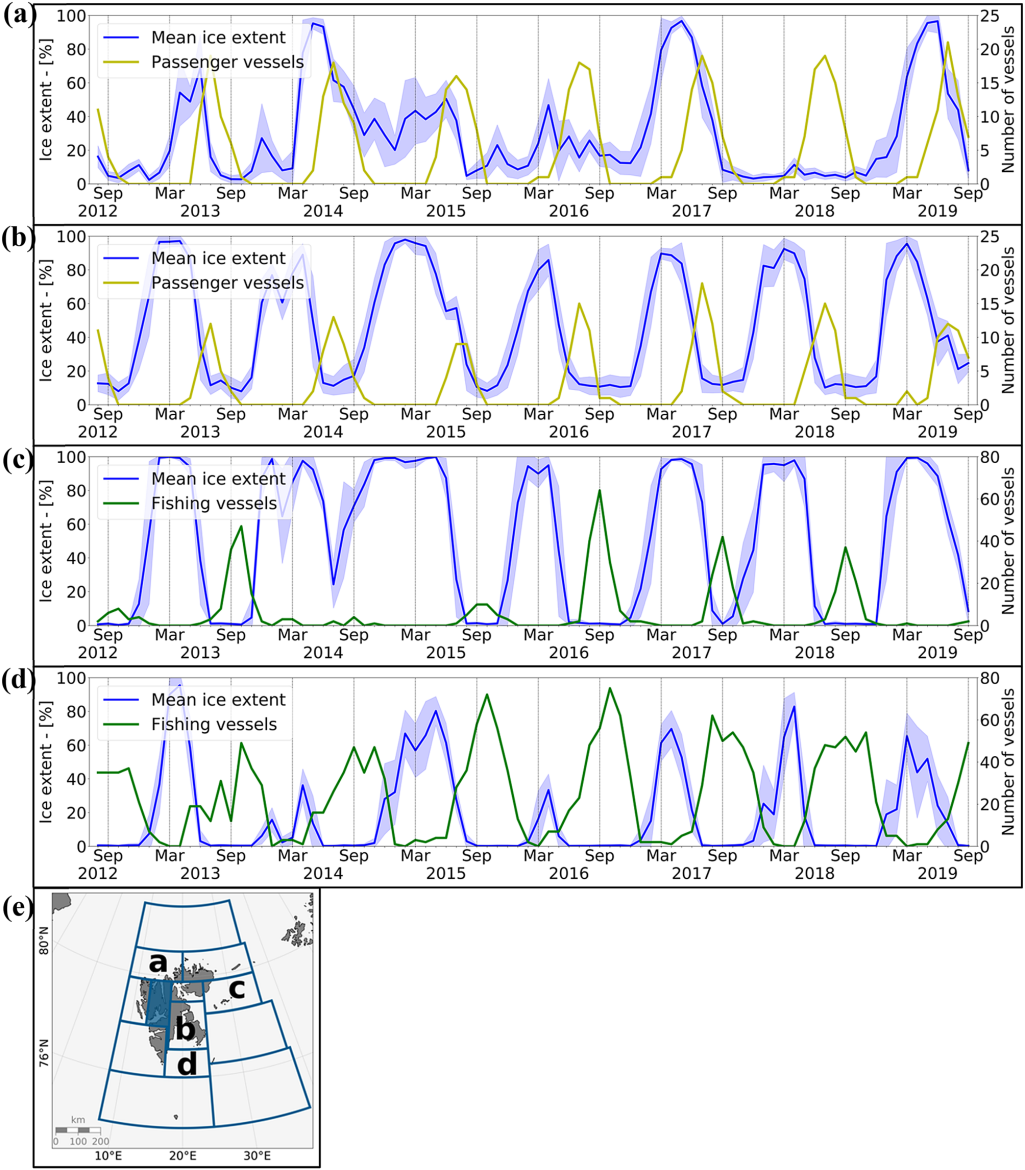
Mean relative sea ice extent, with monthly standard deviation and (**a**) passenger vessels northwest of Svalbard, (**b**) passenger vessels in Storfjorden, (**c**) fishing vessels northeast of Svalbard and (**d**) fishing vessels in Storfjordrenna. (**e**) Map of Svalbard with boundaries of 12 zones, the shaded area was excluded from the analysis due to uncertainty of the sea ice data when in proximity to land. (**a**–**d**) highlight the location of the time series are shown here. Figures generated with python 3.7.x (https://www.python.org/).

The analyses have shown a slight overall increase in maritime activity in the SFPZ and, perhaps more remarkable, a stretching of operational seasons for both sectors. In addition to this, the data show changes in the geographical areas where operators navigate. While expedition cruise vessels are seeking more opportunities to circumnavigate the archipelago, fishing activity is moving northwards. These changes in navigational areas seem to follow the inter- and intra-annual sea ice variability between August 2012 and September 2019 (Fig. 4). Around Svalbard, the sea ice is present in the east and north over the winter and spring months. The west coast is ice-free throughout the entire study period (not accounting for ice in fjord areas) and the ice edge rarely reaches south of 76°N. All interviewees confirm that they experience strong interannual variation. To analyze spatial patterns, we divided the study region into 12 different zones around the archipelago (Fig. 4e). For each zone, the monthly mean sea ice extent and the monthly number of vessels were extracted over the study period. Figure 4 illustrates two representative examples per vessel category: for passenger vessels we show the northwest (a) and Storfjorden (b); for fishing vessels we selected the northeast (c) and Storfjordrenna (d). All zones can be found in the Supplementary material online.

Circumnavigating Svalbard is popular for expedition cruise vessels and is progressively offered by companies. However, the northeast of the archipelago is only ice-free a few months per year and presents strong annual sea ice variability. Highly variable sea ice distribution makes circumnavigation an uncertain activity and challenges itinerary planning^18^, for which operators mainly use historical and daily ice charts from the Norwegian Meteorological Institute^32^. It is predicted that the ice cover around Svalbard will continue to decline^15^, which enables circumnavigation earlier in the season. However, an ice-free Arctic over the summer might become less attractive for tourists, as the unique icy landscapes and endemic wildlife might become more difficult to reach^31^. The maps illustrate that some expedition operators seem to be willing to sail to high latitudes to reach the ice edge, as the map for September 2013 illustrates (Fig. 3). However, while sea ice remains one of the trademarks of Arctic expedition cruising, it is also something that operators try to avoid. In the Storfjorden area (Fig. 4b), we see that in the years with a high sea ice cover (July 2015 and July 2019), fewer expedition vessels visited the area than during the years with low ice cover.

For oversea cruise ships, the main destination in Svalbard is Longyearbyen (Fig. 1), where they stay for ten hours before continuing their itinerary, as explained by a representative of this industry. The west coast of Svalbard remains ice-free year-round, and hence, for this region itinerary planning is hardly affected by sea ice variability. However, this sector has experienced restrictions in sailing areas due to expansion of the ban on the use of HFO. The expansion included Kongsfjorden and Magdalenefjorden (Fig. 1), which prior to 2015 could be visited by conventional cruise vessels.

For fishing activity, the observations illustrate that the majority of activity takes place in the south of the SFPZ throughout the study period (Fig. 3). The increasing number of fishing vessels in the SFPZ could be connected to the northward migration of cod^23^. However, a fisheries representative argued that other fisheries are not moving north as predicted. Sea ice remains an important constraint for fishing vessels, mostly in the east of Svalbard north of 77°N. In all 12 zones, fishing vessels are present when the sea ice cover is below 20%, as illustrated for Storfjordrenna and the northeast (Fig. 4). However, in the northeast (Fig. 4c) little sea ice was present between August and November 2015 and yet few vessels sailed there, which illustrates that fisheries mobility patterns are the consequence of a complex combination of factors, such as the accessibility (sailing time from the mainland), the location of the fishing grounds^20,21^ and the challenges related to Arctic navigation.

Our analyses show that the strong sea ice variability around Svalbard between 2012 and 2019 has impacted destination plans for expedition cruise vessels and partly determined the operational waters for fishing vessels in the north and east of the SFPZ. These increases in activity have implications for safe navigations, which, among others, calls for more accurate sea ice information in remote areas. The following section will discuss the potential implications of these changing mobility patterns to ensure safe and sustainable development of maritime activities around Svalbard.

## Discussion

Climate change impacts the Arctic environment, and Svalbard is strongly impacted. Overall, the strong sea ice retreat results in more ice-free summers. Consequently, new and remote areas are becoming accessible to navigation. This leads to increased maritime traffic, and a stretching of operational seasons and areas, as illustrated here for passenger and fishing vessels in Svalbard waters between 2012 and 2019. Sailing to the northeast of the archipelago has grown in interest, which pushes cruise operators to test limits and sail close to the ice edge until circumnavigation is possible. At the same time, fishing vessels expand their operations in these remote, and more frequently ice-free waters. These developments raise concerns about the possibilities for safe and sustainable navigation and put pressure on supporting infrastructure and regulatory arrangements^33^.

Supporting infrastructure for safe maritime activity consist of, among others, safety and emergency response capacities, and communication and information systems. While SAR capacity around Svalbard is dimensioned to assist a fishing vessel in distress^34^, the main emerging challenges are (1) to adapt to vessels with growing passenger capacity^35^ (an issue that was emphasized with a cruise ship evacuation along the Norwegian coast, March 2019^36^) and (2) to access increasingly remote areas, year-round (a challenge that was stressed after a fishing vessel ran aground in Hinlopen Strait (Fig. 1), December 2018^37^). Increased activity and expanding operational seasons raise concerns for environmental protection and human safety, and pushes the limits of SAR infrastructure in place^38^. In its recent Svalbard strategy^10^, the Norwegian government emphasized the need to assess preparedness and safety challenges arising from increased shipping activity in the Arctic, and currently investments are made to improve the material infrastructure. Despite the under-dimensioning in state preparedness around Svalbard, from a pan-Arctic perspective it might be argued that Svalbard has a relatively well-developed safety infrastructure, and it can be assumed that safety preparedness in other Arctic regions is to an even larger extent a major responsibility of the operators^39^.

With respect to communication and information infrastructure, there are efforts to improve communication in these waters, through prioritizing high frequency radio coverage up to the North Pole^40^. In addition, the improvement of Arctic weather and sea ice forecasting services has gained a lot of attention since the Year of Polar Prediction^41^. The strong seasonal and inter-annual sea ice variability around Svalbard in parallel with increasing maritime activities has a demand-pull effect on achieving the need for accurate and reliable sea ice services^42,43^. End-users operating around Svalbard emphasize the need for publicly accessible ice charts to be available daily, even more so when sailing in the north and east of the archipelago. For the moment, ice charts are available on weekdays, while automated charts are published during the weekends, but there is no warranty on their accuracy^44^. Therefore, it is imperative to put in place opportunities for improvements towards higher resolution, accurate charts to better support maritime activity in the Arctic^45^. It is also essential to provide products with low-bandwidth solutions in order to be easily accessible to a wider range of vessels sailing at higher latitudes. Such sea ice information needs are dependent on the type of use^46^; while fishermen primarily need information about the location of the ice edge, cruise vessel operators desire more detailed information about the type of sea ice in their navigational areas.

Several governance regulations have been implemented or expanded in recent years to mitigate risks and limit human impact, such as IMO’s Polar Code, which acts as a baseline for requirements on various levels (design, construction, equipment, operational, training and environmental protection matters) for navigation in polar waters^9^. However, specific regulations are needed to adapt to local development of maritime activities. The HFO ban in protected areas around Svalbard acts as geographical restriction for large ships and could serve as an example for other Arctic regions. Within the IMO and the Arctic Council, there is growing discussion to ban HFOs throughout the Arctic^47^, which would be an important step forward in sustainable navigation. With regard to the predicted growth in cruise tourism in the Arctic^5,11^, in January 2020 the Norwegian Maritime Authority implemented specific regulations for passenger ships sailing in protected territorial waters, which account for 87% of all territorial waters around Svalbard^48^. These additional regulations include the prohibition of discharge of sewage and gray water within 500 m off land and the prohibition of sea traffic in bird sanctuaries between May 15 and August 15^49,50^. Hence, while maritime development around Svalbard will partly be shaped by future ice conditions, its sustainability and trends will largely depend on international and national policy developments^16,51^.

Despite ongoing work to enhance supporting infrastructures and sharpen regulations, and thus the growing controllability of maritime traffic around Svalbard, this might invite for a further increase in activities in the decades to come. Indeed, some argue that environmental concerns are unlikely to lead to very strong restrictions and limitations of exploitation of Arctic resources^52^. It will be highly relevant to follow the implementation of the Norwegian government’s recently updated ocean strategy as well as its High North strategy^10,53^, and what these imply in terms of safety and sustainable maritime development around Svalbard. Despite ongoing efforts at national and international levels, navigation in these areas will always remain challenging due to persisting seasonal darkness, remoteness and potentially hazardous weather and sea-ice conditions. Still, the development of SAR capacity and communication and information systems are ascending on the political agenda, not only because of the increasing local and destination traffic as illustrated in this paper, but also due to the projections of Svalbard’s strategic future location along a potential Arctic trade route over the North Pole^54^.

## Conclusion

This paper has provided insights into the development of maritime activity around Svalbard, and has presented an approach that will be relevant to understand transformations in other Arctic regions. The combination of vessel tracking data through AIS and sea ice data have enabled us to provide novel regionally contextualized insights about trends in Arctic shipping activity and how these relate to climate change. To conclude, we outline complementary ways to achieve a comprehensive understanding of maritime change and its implications in Arctic regions, serving to guide safety and sustainable development.

We approached maritime change by focusing on unique vessel activity per month, which has primarily provided an enhanced understanding of expanding sailing areas and seasons. Naturally, there are several other possible approaches with AIS data, such as measuring the total distance sailed by all vessels per category, which would be crucial information in the dimensioning of safety and response capacity.

In the Arctic, investments in satellites recording AIS signals are growing, making higher temporospatial resolution data accessible. This increases the possibilities to analyze mobility patterns in determined areas over shorter time periods (e.g. weekly timeframes), which would elucidate vessel’s behavior in proximity to the ice edge and in the marginal ice zone.

Developments in the marginal ice zone are very relevant to follow, since this dynamic zone between open ocean and dense pack ice will continue to expand in the summer^55^ and is predicted to be an interesting place for maritime activity^31^, but highly challenging in terms of risk and safety.

We were particularly interested to understand changes in the cruise tourism and fisheries sectors, which provided us with the possibility to reflect upon some of the dynamics that are at play in each of these sectors. Naturally, a more comprehensive picture of maritime change would include several sectors, such as cargo shipping, research, military and surveillance activities. Past trends can be understood by using the wealth of AIS data that have been generated in the past years. However, not all vessels carry AIS transponders, and a mapping of all vessels is vital for understanding the overall safety challenges in the Arctic, especially since more and more pleasure yachts are exploring the area.

A comprehensive study of maritime activity trends increases in value when *past* trends are analyzed in line with *projected* maritime change, which might include a focus on foresight and scenario studies and a scrutiny of government and business strategies for industrial innovation and development for any particular region. Such a comprehensive understanding can lead to a better overall picture of maritime transformations in Arctic regions and the risks and uncertainties these entail.

## Methods

### Sea ice data source and limitations

This study used daily sea ice concentration data from ARTIST Sea Ice (ASI) algorithm applied to Advanced Microwave Scanning Radiometer (AMSR2) from the open dataset of the University of Bremen^56^. AMSR2 has been delivering sea ice concentration data since August 2012^56^, which sets the beginning of the study period. AMSR2 has a horizontal resolution of 6.25 km and has a specific grid over the Earth. For each grid point the average sea ice concentration is calculated by ASI and is associated with coordinates representing the center of the grid point. However, this resolution presents some limitations close to land, where coastal reflection was detected as sea ice. This limitation does not affect the study, as ice cover in fjords is not considered in this study.

### AIS source

AIS is a precise satellite-based source of data continuously recording spatial ship traffic. Initially established by IMO as a safety measure at sea for large commercial ships to avoid collision, AIS first became effective by the end of 2004^57^. Since, AIS has been growing in interest for coastal states to ensure safety and used more frequently as a tool to establish sustainable management of maritime activities^58^. Several studies have analyzed mobility patterns with AIS data on a global scale^59^ and in the Arctic^11,60^. In this study, AIS data from vessels operating around Svalbard between August 2012 and September 2019 were obtained from the Norwegian Coastal Administration (NCA). Monthly files had the following information; Maritime Mobile Service Identity (MMSI) number, IMO number, name of vessel, vessel length, timestamp (UTC), longitude, latitude, speed over ground, course over ground, true heading (determines geographical direction in degree 0–360), navigation status, message number (class A: 1–3; class B: 18,19) and source (satellite/ground). Both commercial and recreational vessels are included in this dataset, which are categorized by class A and class B respectively. AIS signals are recorded every 2 s to 10 minutes^61^, depending on vessel class and navigational status, and whether signals are detected by satellite or ground receptors. The NCA uses AIS data from four satellites to monitor vessel traffic off Norwegian and Svalbard coasts. As AIS data are publicly available (upon request) not all vessel information is provided. Therefore, to obtain ship type information on vessels sailing in the SFPZ a special request was done; this second dataset excluded vessels under 15 m. In this study, the IMO number was used to merge the AIS and vessel type information data. We processed over 120 million AIS signals and used the IMO number, timestamp (UTC), longitude, latitude, and vessel type to determine mobility patterns around Svalbard.

### AIS limitations

AIS data are not homogenous time series due to satellite limitations, technology innovations and transponder problems. Satellites are crucial to receive signals beyond the limits of coastal receptors; however, signals will not be detected if the satellite is out of reach or if the vessel is near a geographical barrier. The investment in new satellites has led to an increase in the frequency of signals between 2012 and 2019. The four AIS satellites in Norway were launched at different periods: AISSat-1 in July 2010, AISSat-2 in July 2014, NorSat-1 and NorSat-2 in July 2017^61^. Multiple satellites allow for a more continuous registration of AIS signals, whereas only one satellite results in gaps in the data when orbiting the globe. Furthermore, AIS data are susceptible to problems related to the AIS transponder, issues stemming from modifications by users, and errors in the transmission, reception, storage and decoding of information^62^. Among these limitations, two in particular affected our analyses: (1) vessel information such as the IMO number filled in by the crew can hold some errors, therefore some vessels were not categorized and therefore excluded from analysis; (2) the poor satellite coverage at high latitudes may limit the position registered and lead to loss of the northernmost positions.

### Measuring maritime activity

To measure maritime activity in SFPZ we counted the number of unique vessels sailing in the area, using the AIS data where each vessel has a unique IMO number. Each IMO number is counted once per month even if the same vessels sailed multiple times through the SFPZ in that time period. The delimitation of the SFPZ is roughly from 72°N to 84°N and 3°W to 38°E (Fig. 1). For each month, these vessels were categorized by passenger vessels (combining oversea cruise ships and passenger vessels), fishing vessels, and all other categories were grouped together, which include bulk carriers, container ships, icebreakers, etc.

### Mapping method

To visualize the relationship between passenger and fishing vessels with sea ice variability, a series of maps were made. These maps of ice concentration and ship tracks were created with python 3.7, using basemap (1.2.0), matplotlib (3.0.2), numpy (1.15.4) and pandas (0.23.4) libraries, and using a Polar Stereographic projection. Each map shows the monthly mean sea ice extent as well as all passenger and fishing AIS signals detected that same month. The monthly mean sea ice concentrations were calculated from the daily AMSR2 grid. In this study, the ice edge is delimited at 15% ice concentration, and below this limit is defined as open water. On the maps, the boundaries of sea ice concentration are at 15%, 30%, 45%, 60%, 75%, 90% and 100% (Fig. 3). The colors associated are from cmocean (2.0)^63^, here the ice colormap was selected from the cmocean gallery. Concerning AIS signals, in order to compare mobility patterns throughout the study period, vessel positions are shown with a frequency of 60 min on the maps. This allowed a better comparison between earlier and later years, due to satellite innovations explained above. In total 86 months were analyzed and ten maps (February, April, July, September and November of 2013 and 2017) are presented in this paper. In this study, 2013 is the first complete year of both AMSR2 sea ice concentration and AIS data. 2017 has a particular high sea ice cover over the summer season and is of particular interest to analyze. The selected months in this paper allowed us to give an overview of both seasonal sea ice variability and mobility patterns. All maps between August 2012 and September 2019 can be found in the Supplementary material online.

### Time series method

For further analysis of the relationship between sea ice variability and mobility patterns around the archipelago, 12 different zones were defined (Fig. 4e). The size and limits of these zones were determined by the sea ice extent and sailing areas of interest for fishing and passenger vessels based on map observations between August 2012 and September 2019. For each zone a time series showed the monthly sea ice extent and the number of unique vessels per month throughout the study period. The AMSR2 grid was used to calculate the monthly mean sea ice cover. Each of the 12 determined zones has a specific number of AMSR2 grid points that was constant throughout the study period. The percentage of relative sea ice cover in each zone was defined as the number of the AMSR2 grid points that were above 15% sea ice concentration. From the daily sea ice cover, the monthly mean and standard deviation were calculated. The mean sea ice cover allowed to visualize yearly sea ice variability whereas the standard deviation gave additional information on monthly and seasonal sea ice variability. To determine the number of passenger or fishing vessels, each unique IMO number is counted once, independently of the number of times the same vessel crosses the zone in that time period. In this paper four zones are visualized; the northwest and Storfjorden with passenger vessels, and the northeast and Storfjordrenna with fishing vessels (Fig. 4). The larger fjords of Isfjorden and Wijdefjorden have been excluded from zones analysis (shaded areas in Fig. 4e), due to uncertainties related to the AMSR2 resolution as explained above.

### Interviews

The first author conducted seven semi-structured interviews in Longyearbyen and in Tromsø (Norway) over the summer and autumn months of 2018. The participants were captains, sea ice researchers and representatives of fisheries and cruise tourism organizations in the Svalbard and Barents Sea area. These key informants were selected based on their role in decision-making and guidance for the fishing and cruise tourism industries operating around Svalbard. The structure of the interviews allowed to cover important themes around the use of sea ice services around Svalbard, but also gave the participants the freedom to add information they believed important as well. Main themes concerned changes in sea ice variability, vessel traffic, the type of sea ice information used in decision-making and the challenges around operating in waters around Svalbard in the future. All interviews were conducted with the participants’ consent, were all held in person, and most interviews lasted around 30 min. The interviews were conducted in English, which did not result in any language barrier as most Norwegians are fluent in English. Handling and management of the qualitative interview data followed the requirements of the Norwegian Centre for Research Data.

## Supplementary information

Supplementary Information.
