# Supplementary material for: Sea ice variability and maritime activity around Svalbard in the period 2012–2019
Source: Sci Rep. 2020 Oct 12;10:17043. doi: 10.1038/s41598-020-74064-2 (PMC7552395; doi:10.1038/s41598-020-74064-2)
Supplement: Supplementary file 1 — Supplementary Information. [file 41598_2020_74064_MOESM1_ESM.pdf]

Supplementary information

Sea ice variability and maritime activity around Svalbard in the period 2012-2019

Alexandra N. Stocker *[a,b], Angelika H. H. Renner [c] and Maaike Knol-Kauffman [d]

[a] Department of Geography, Umeå University, Umeå, Sweden; [b] University Center of the Westfjords, Isafjordur, Iceland; [c] Institute of Marine Research, Tromsø, Norway; [d] Norwegian College of Fishery Sciences, University of Tromsø - The Arctic University of Norway, Tromsø, Norway

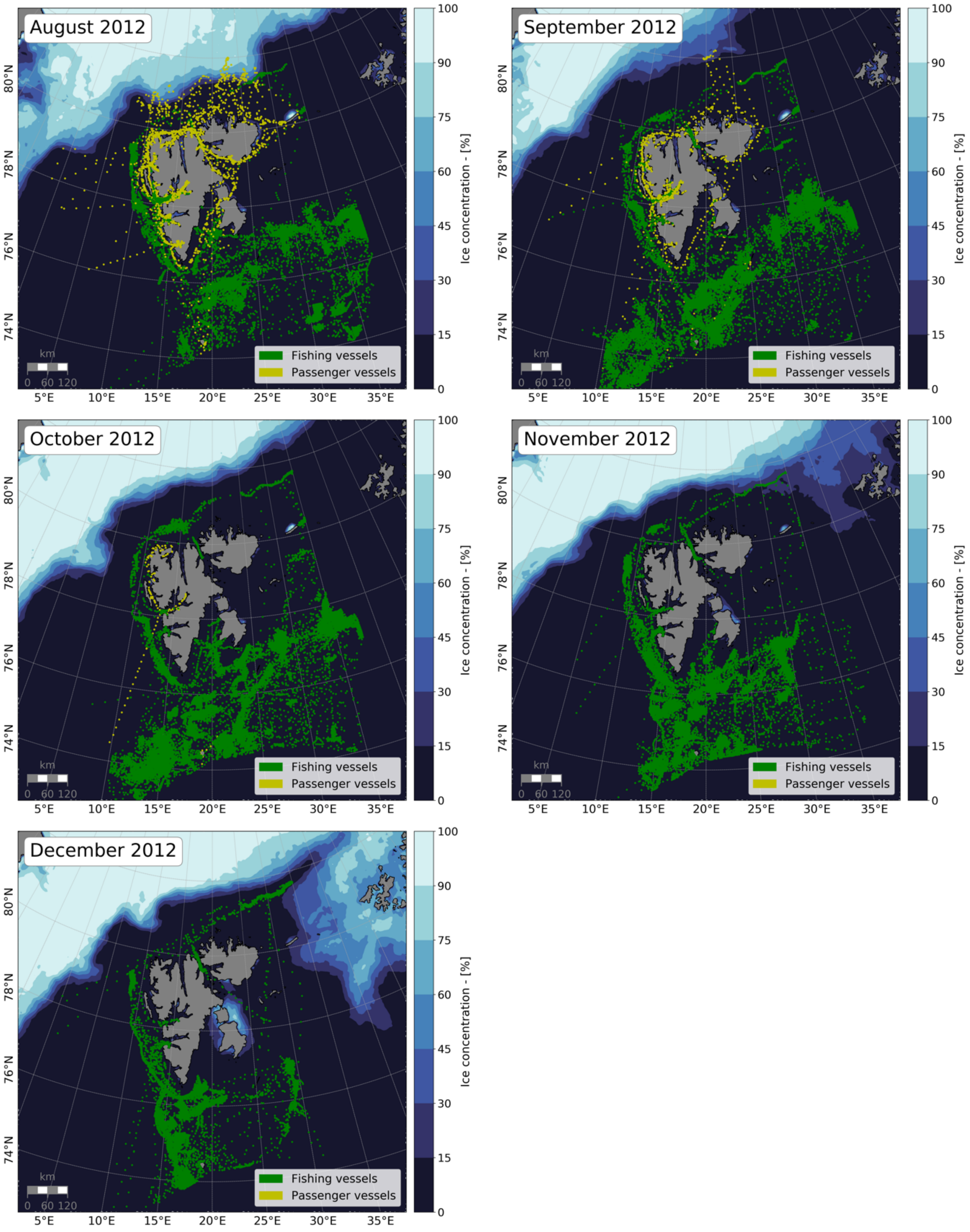

Figure S1: Average sea ice concentration, and fishing (green) and passenger (yellow) vessel positions. August - December 2012. Figures generated with python 3.7.x (https://www.python.org/).

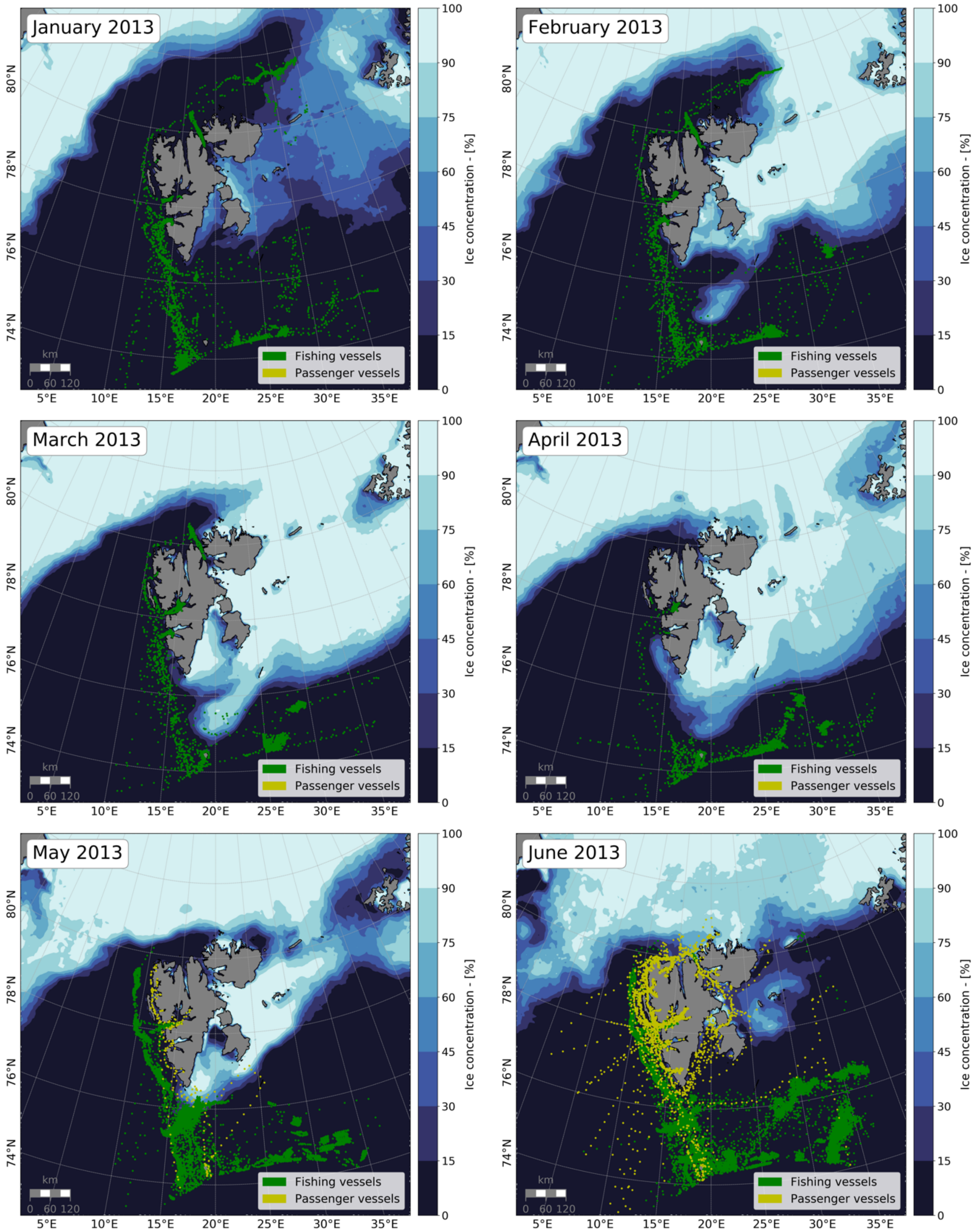

Figure S2: Average sea ice concentration, and fishing (green) and passenger (yellow) vessel positions. January - June 2013. Figures generated with python 3.7.x (https://www.python.org/).

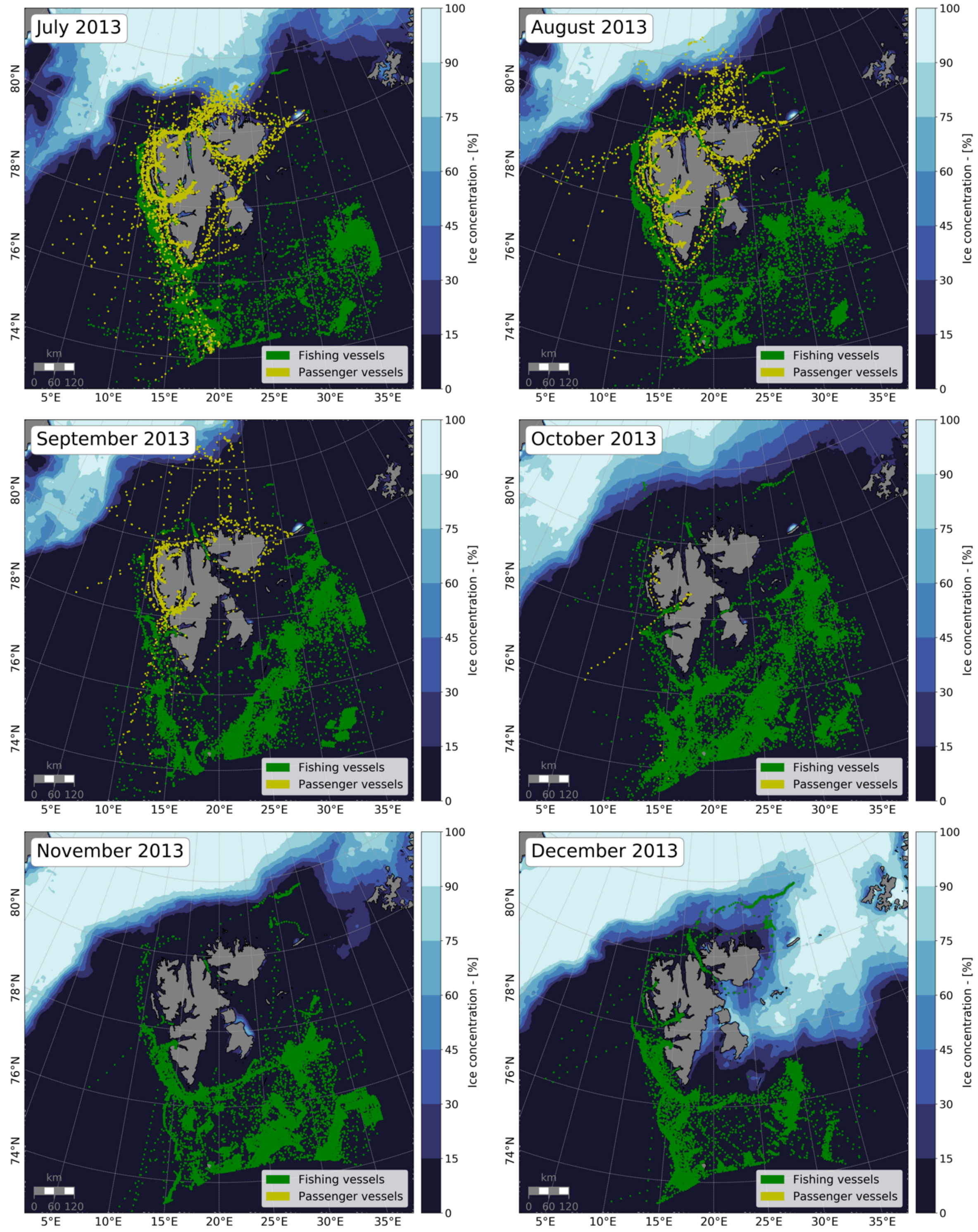

Figure S3: Average sea ice concentration, and fishing (green) and passenger (yellow) vessel positions. July - December 2013. Figures generated with python 3.7.x (https://www.python.org/).

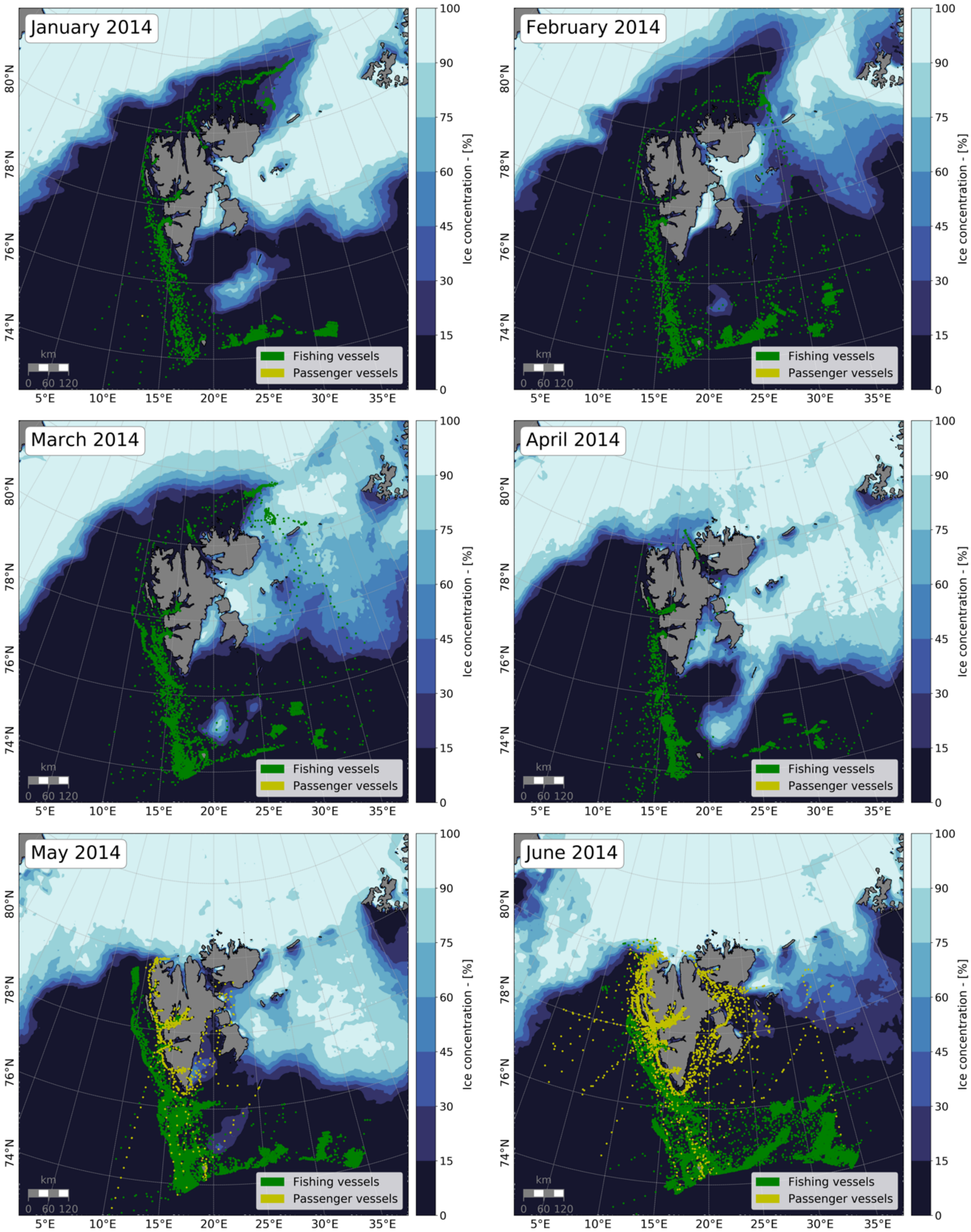

Figure S4: Average sea ice concentration, and fishing (green) and passenger (yellow) vessel positions. January - June 2014. Figures generated with python 3.7.x (https://www.python.org/).

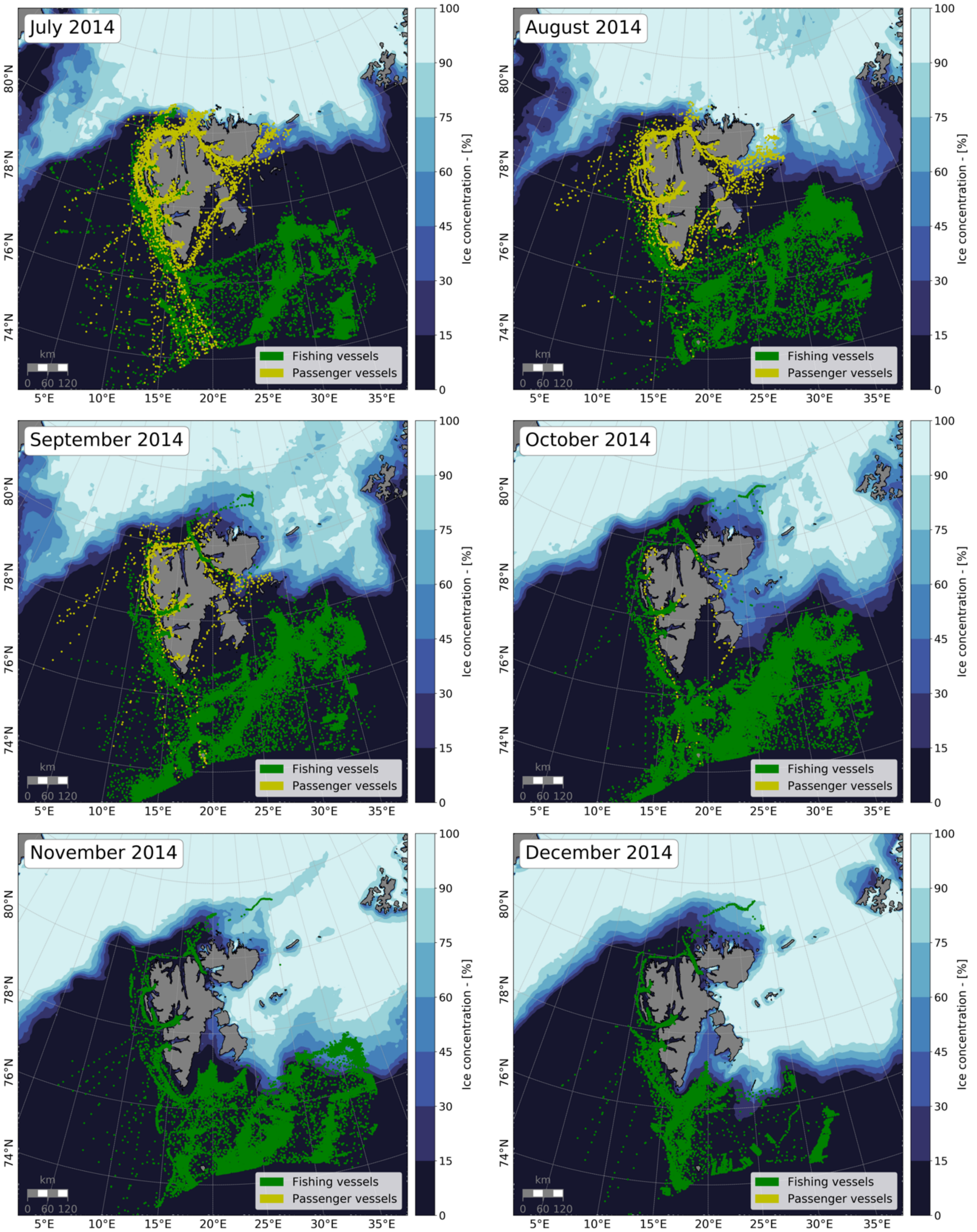

Figure S5: Average sea ice concentration, and fishing (green) and passenger (yellow) vessel positions. July - December 2014. Figures generated with python 3.7.x (https://www.python.org/).

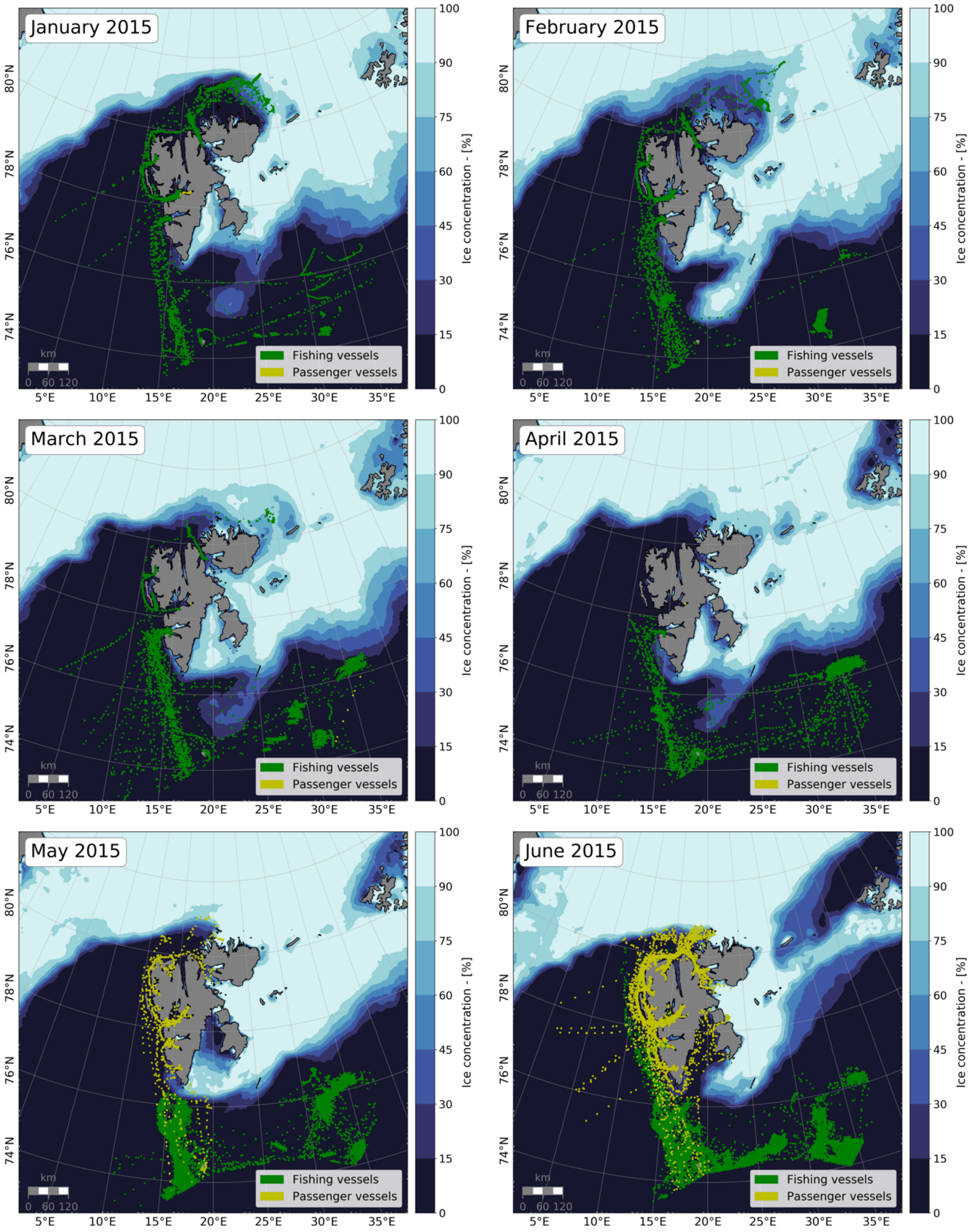

Figure S6: Average sea ice concentration, and fishing (green) and passenger (yellow) vessel positions. January - June 2015. Figures generated with python 3.7.x (https://www.python.org/).

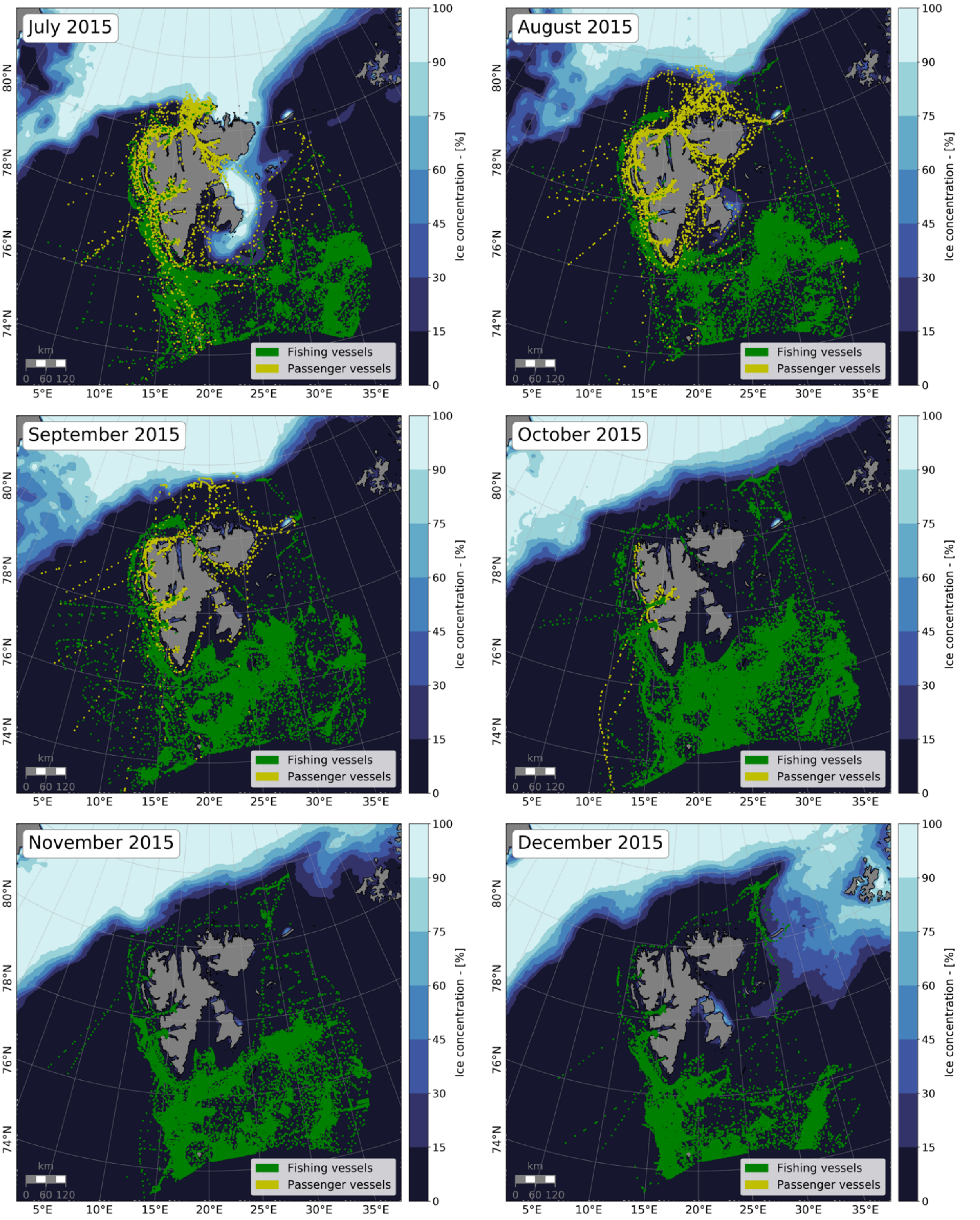

Figure S7: Average sea ice concentration, and fishing (green) and passenger (yellow) vessel positions. July - December 2015. Figures generated with python 3.7.x (https://www.python.org/).

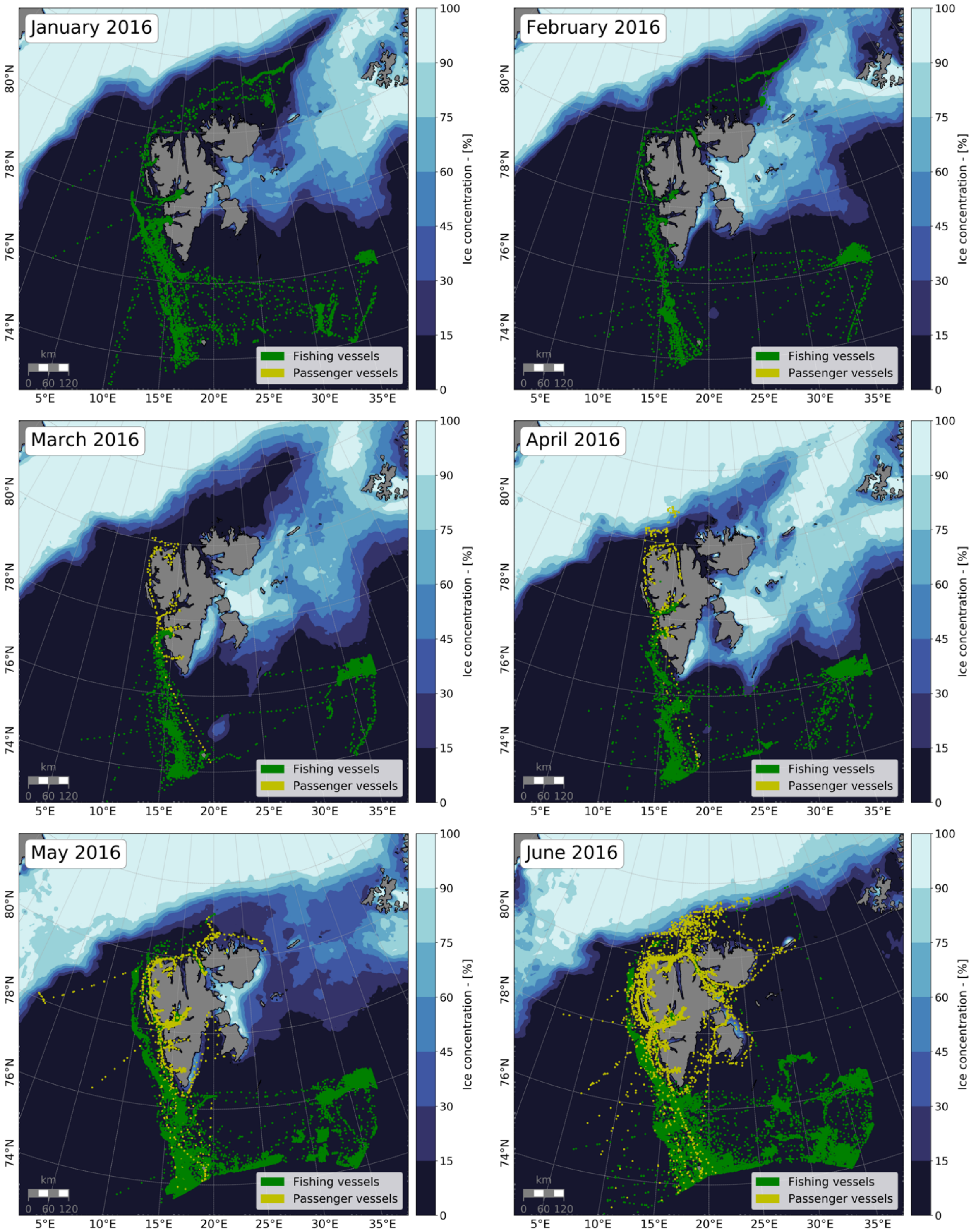

Figure S8: Average sea ice concentration, and fishing (green) and passenger (yellow) vessel positions. January - June 2016. Figures generated with python 3.7.x (https://www.python.org/).

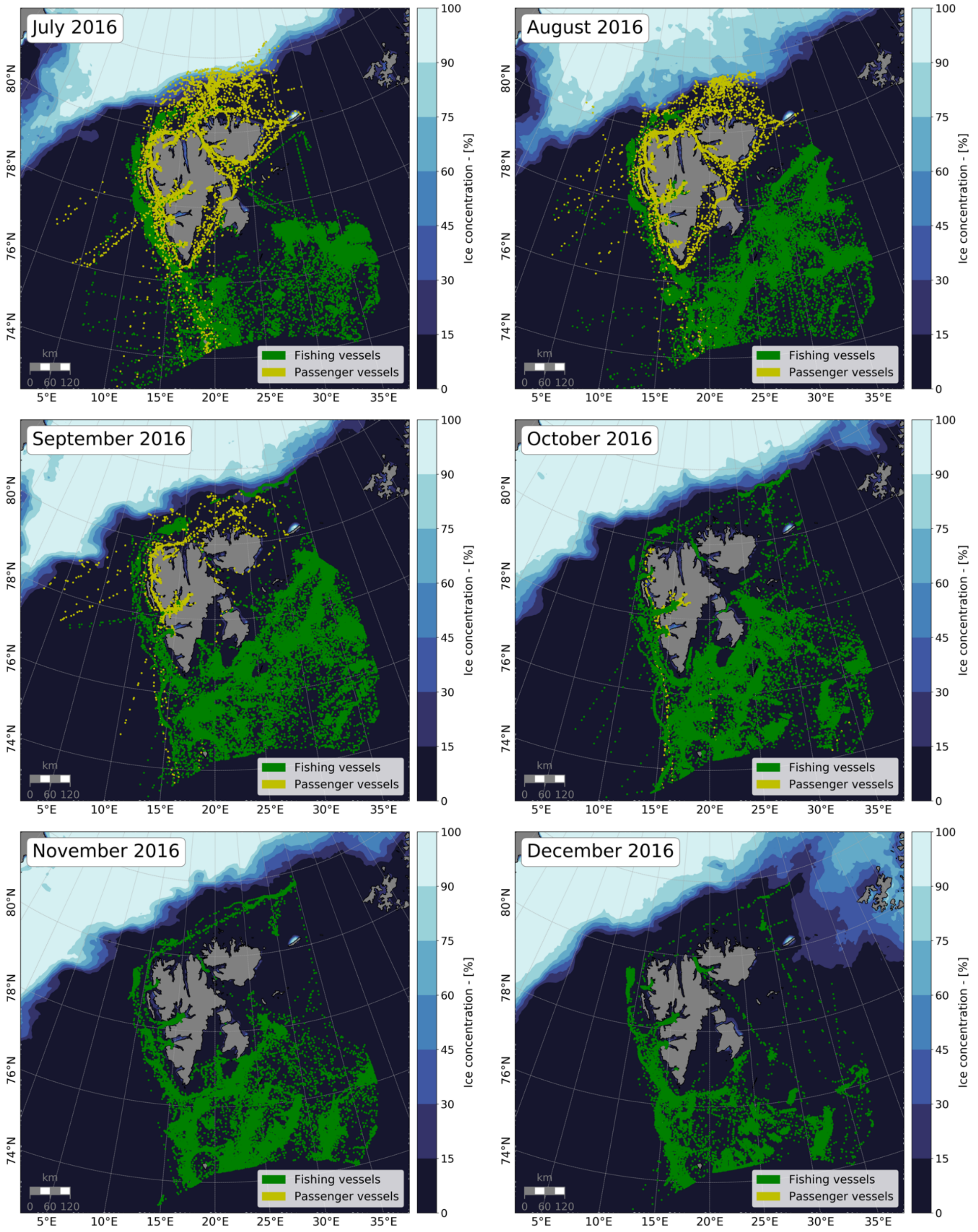

Figure S9: Average sea ice concentration, and fishing (green) and passenger (yellow) vessel positions. July - December 2016. Figures generated with python 3.7.x (https://www.python.org/).

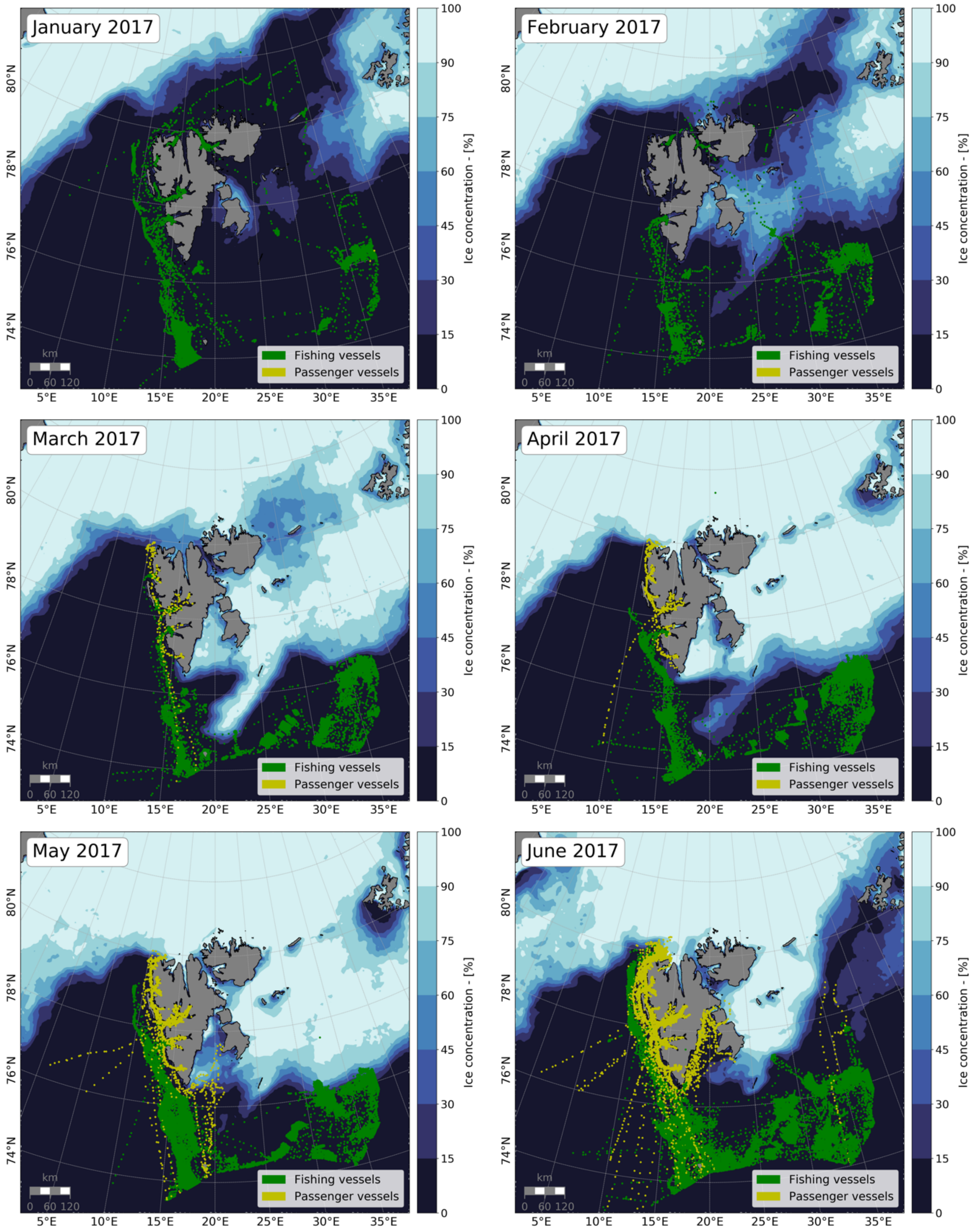

Figure S10: Average sea ice concentration, and fishing (green) and passenger (yellow) vessel positions. January - June 2017. Figures generated with python 3.7.x (https://www.python.org/).

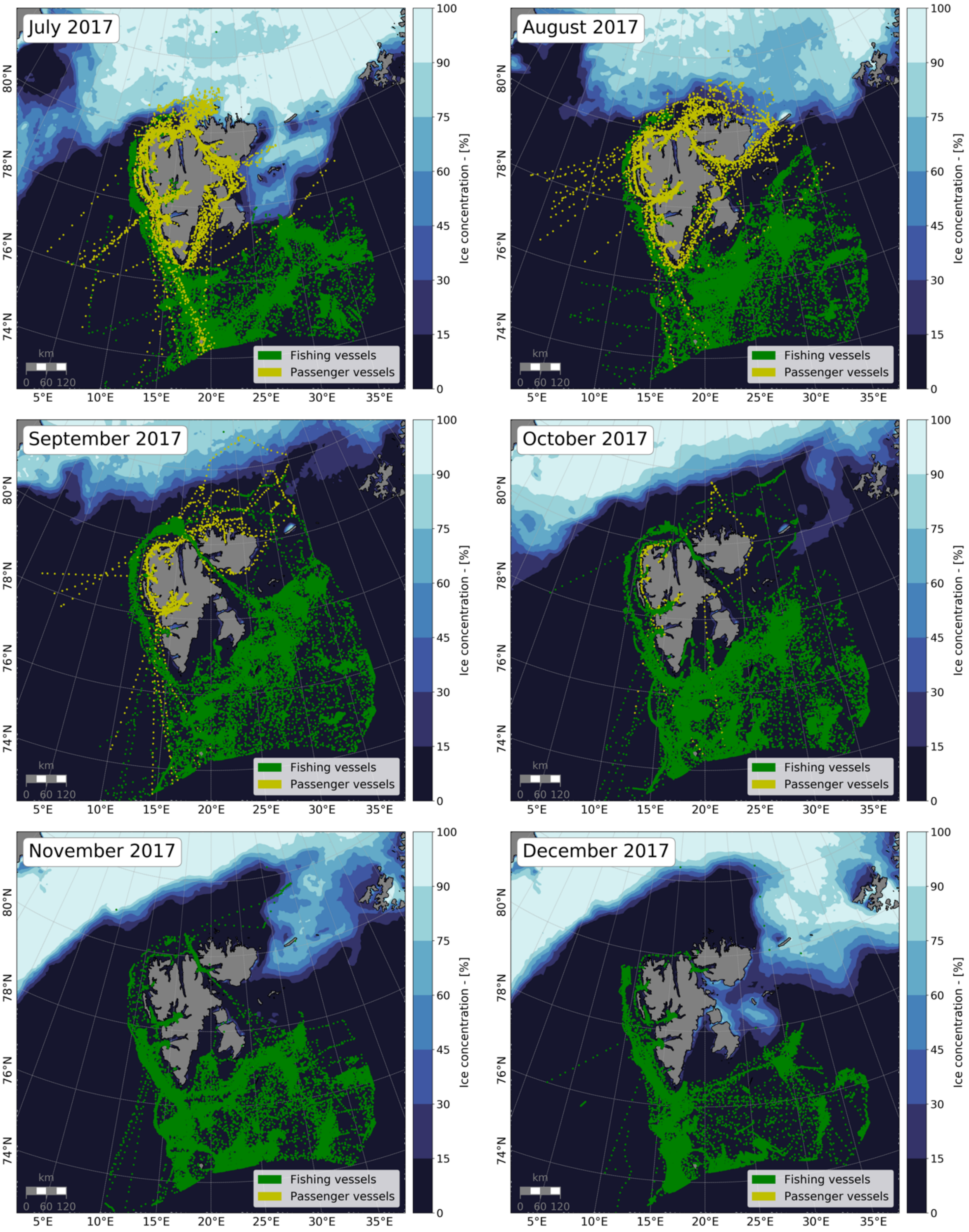

Figure S11: Average sea ice concentration, and fishing (green) and passenger (yellow) vessel positions. July - December 2017. Figures generated with python 3.7.x (https://www.python.org/).

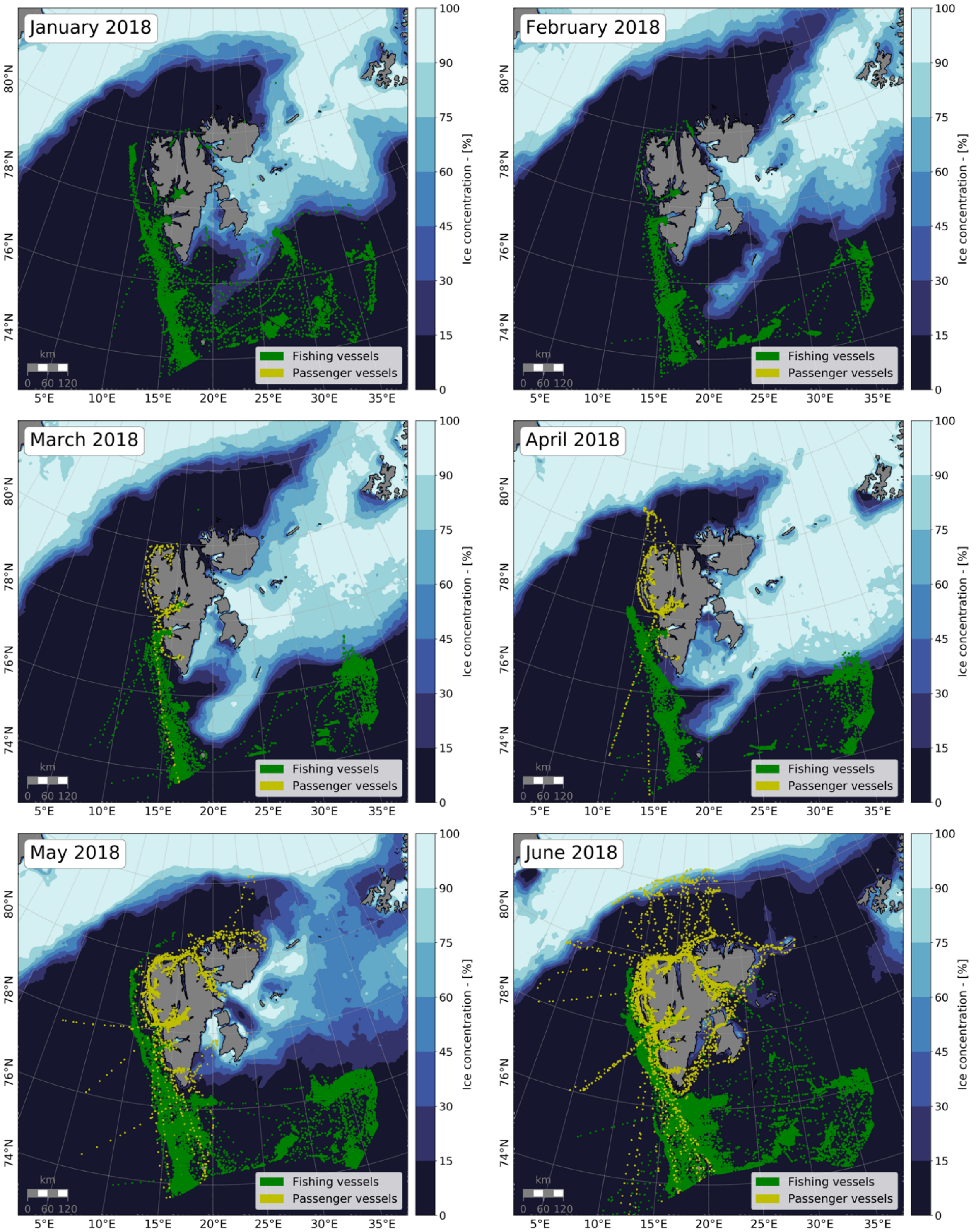

Figure S12: Average sea ice concentration, and fishing (green) and passenger (yellow) vessel positions. January - June 2018. Figures generated with python 3.7.x (https://www.python.org/).

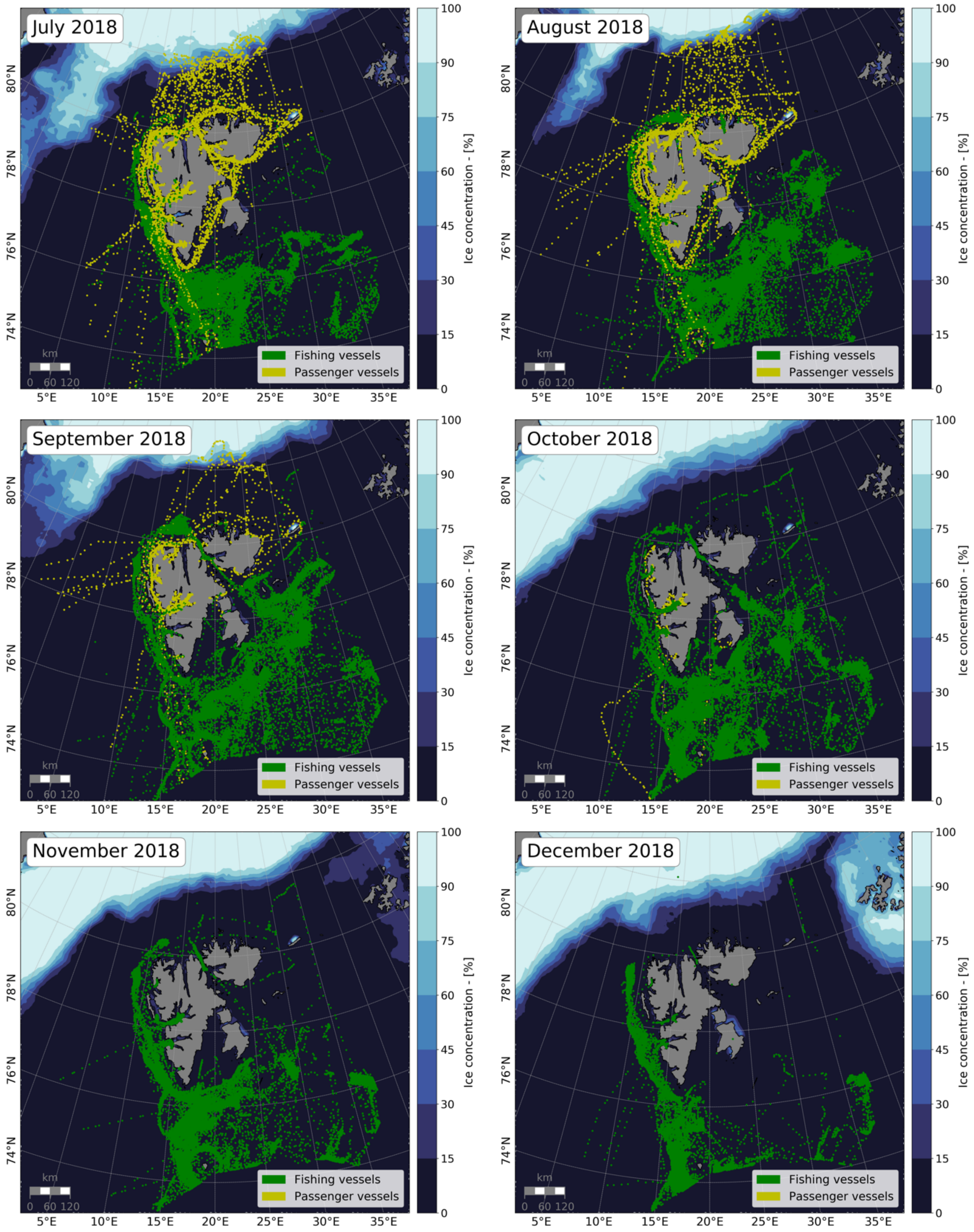

Figure S13: Average sea ice concentration, and fishing (green) and passenger (yellow) vessel positions. July - December 2018. Figures generated with python 3.7.x (https://www.python.org/).

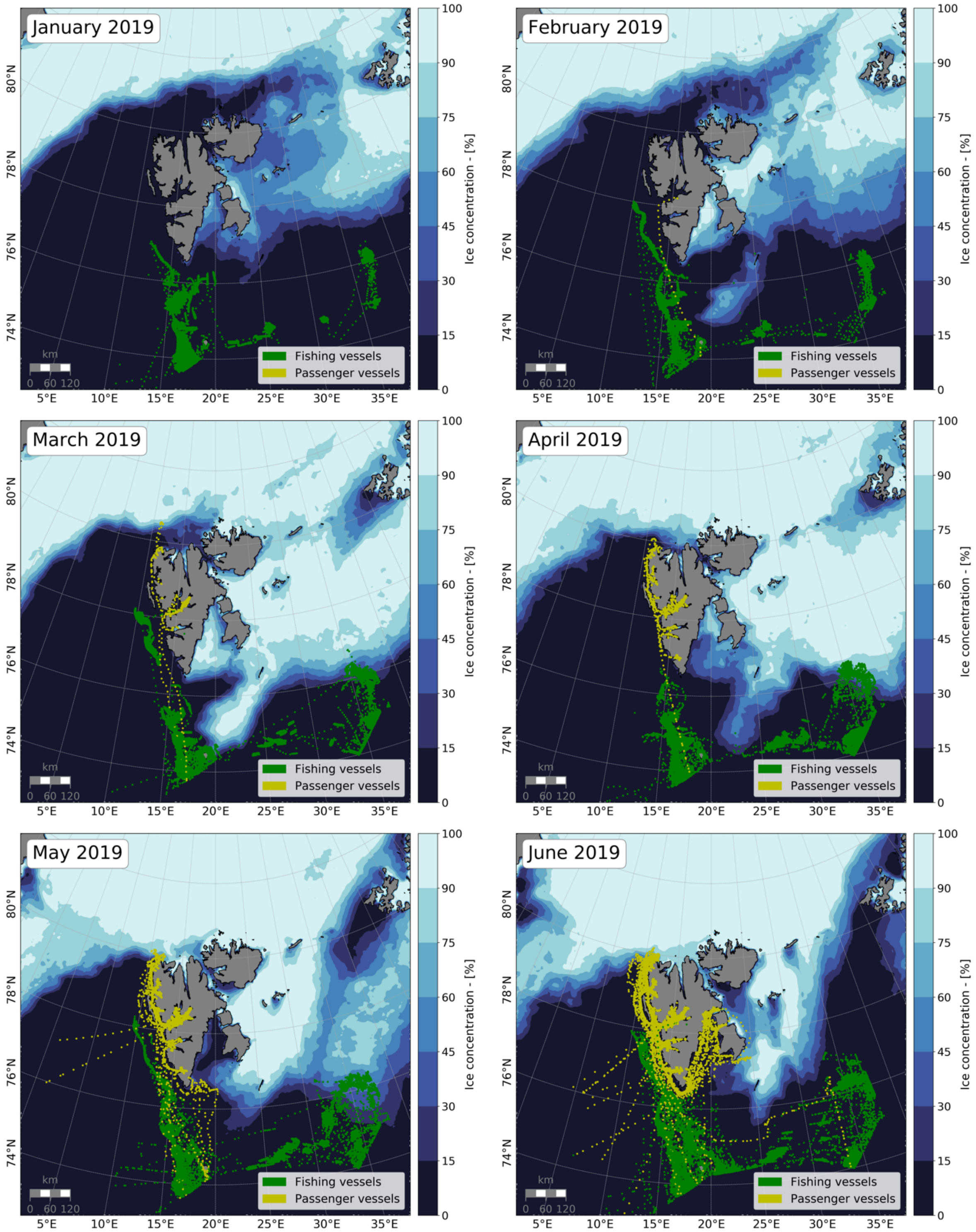

Figure S14: Average sea ice concentration, and fishing (green) and passenger (yellow) vessel positions. January - June 2019. Figures generated with python 3.7.x (https://www.python.org/).

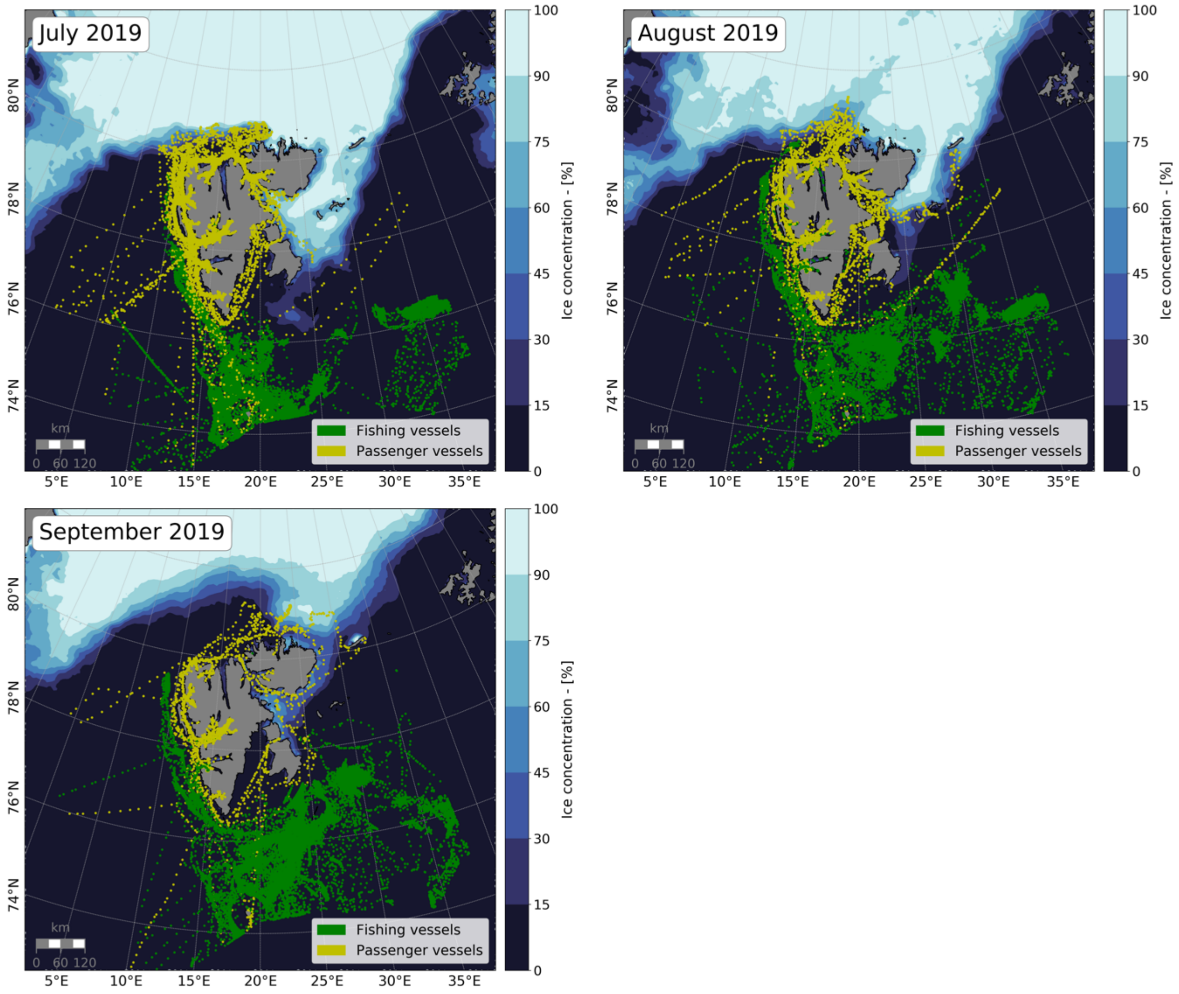

Figure S15: Average sea ice concentration, and fishing (green) and passenger (yellow) vessel positions. July - September 2019. Figures generated with python 3.7.x (https://www.python.org/).

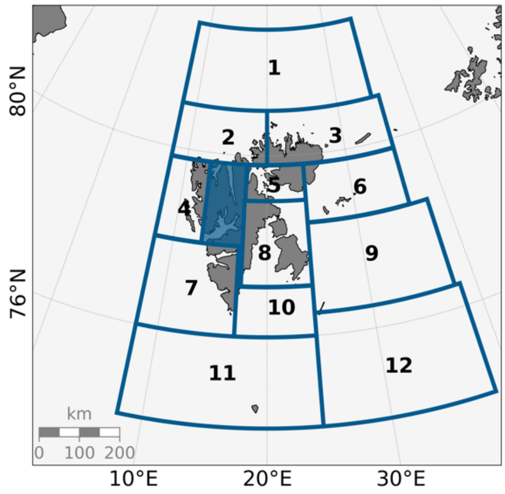

Figure S16: Map of Svalbard with boundaries of 12 determined zones. The shaded area is excluded from the analysis due to the uncertainty of sea ice data when in proximity to land. Figures generated with python 3.7.x (https://www.python.org/).

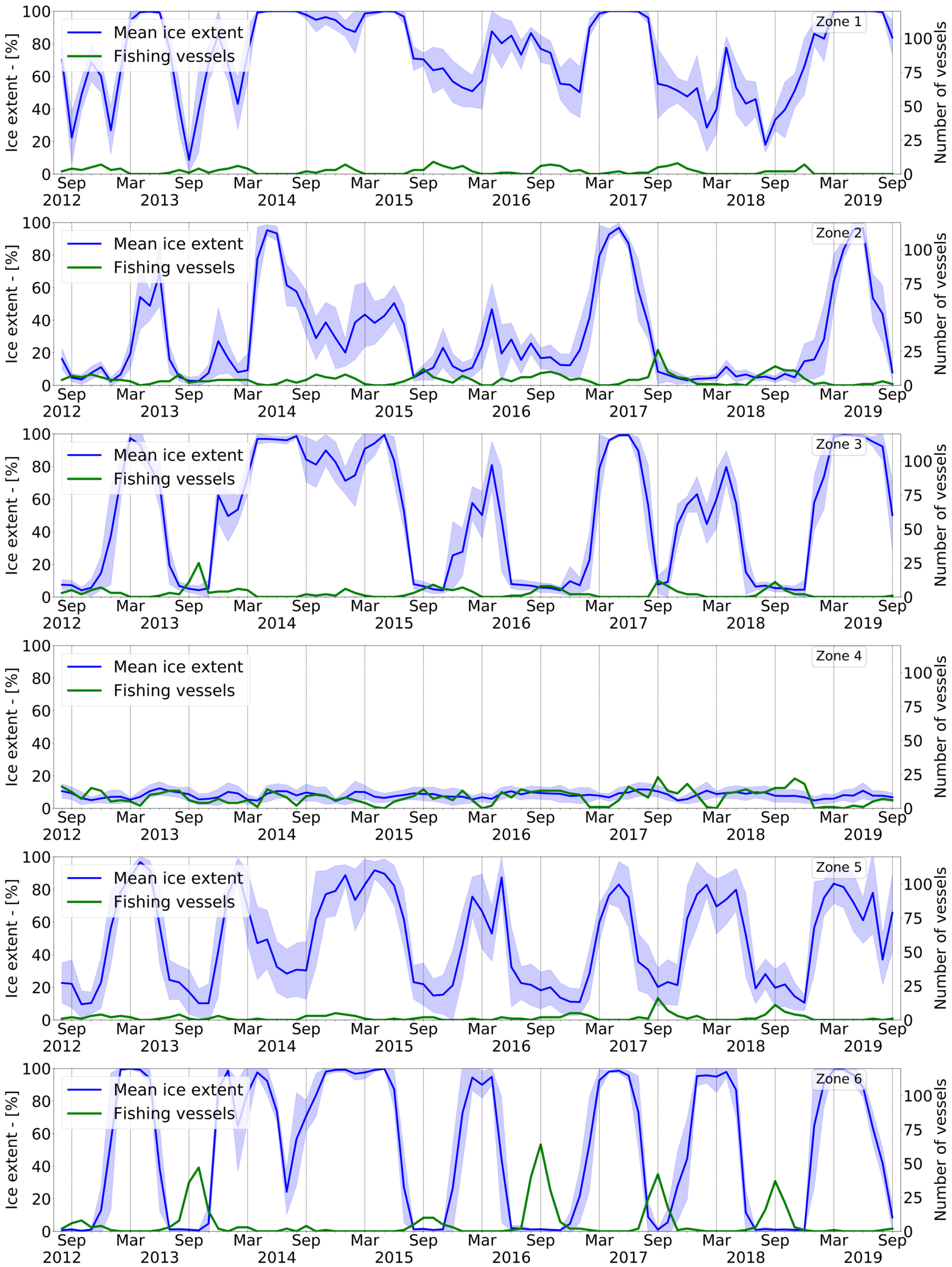

Figure S17: Time series of relative mean sea ice extent, with monthly standard deviation and number of fishing vessels in Zone 1 to Zone 6 (see Figure S16).

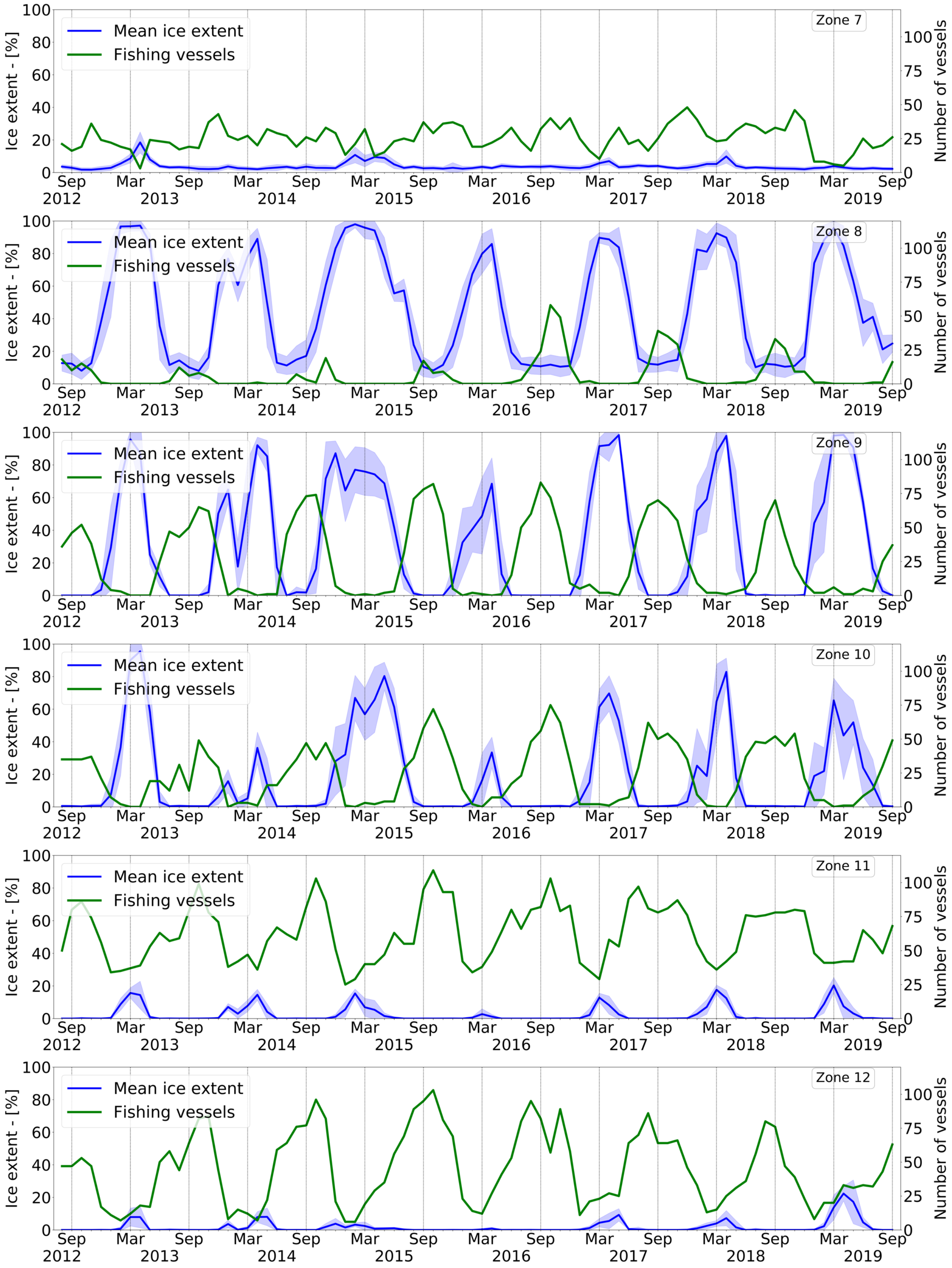

Figure S18: Time series of relative mean sea ice extent, with monthly standard deviation and number of fishing vessels in Zone 7 to Zone 12 (see Figure S16).

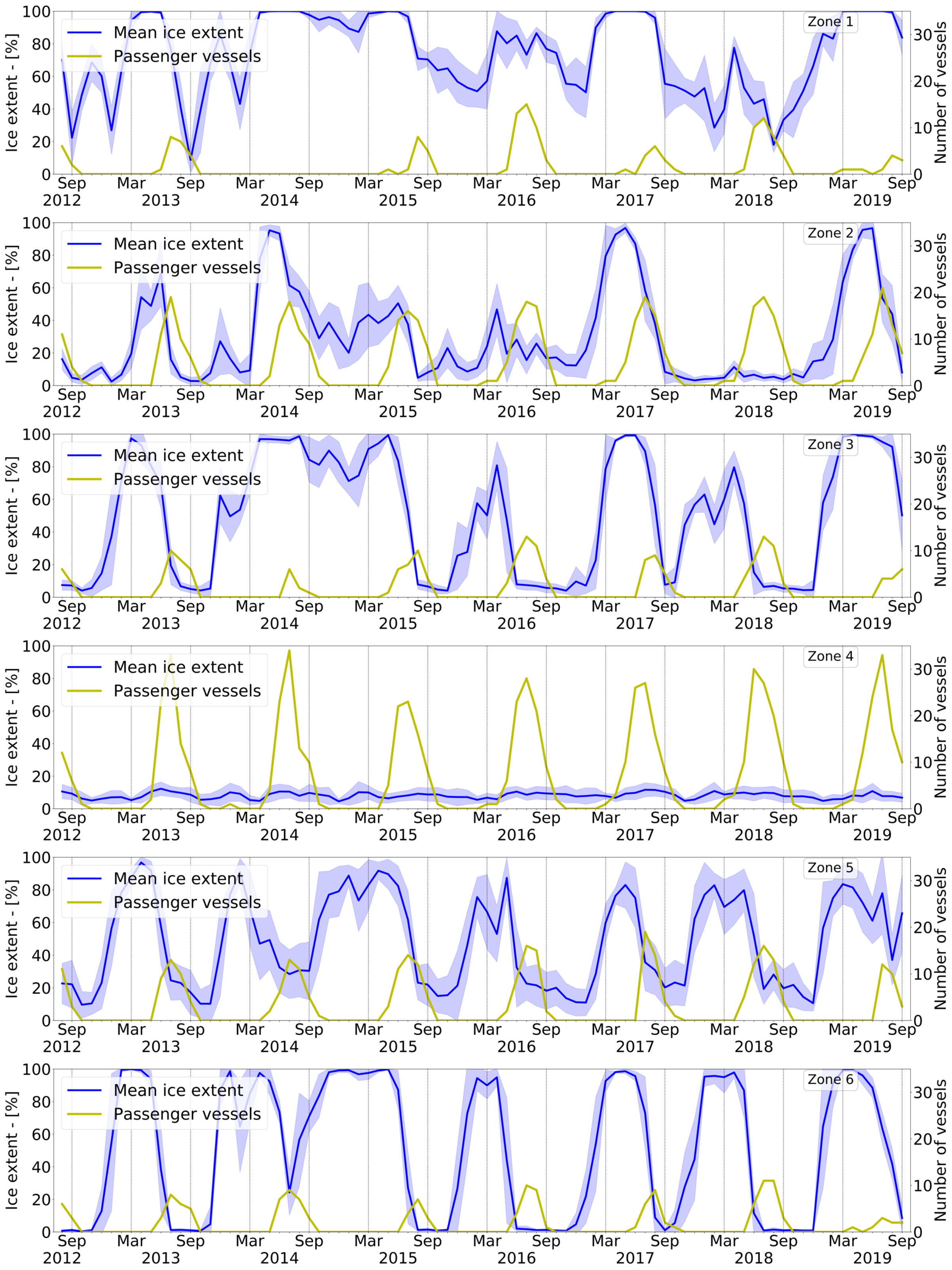

Figure S19:Time series of relative mean sea ice extent, with monthly standard deviation and number of passenger vessels in Zone 1 to Zone 6 (see Figure S16).

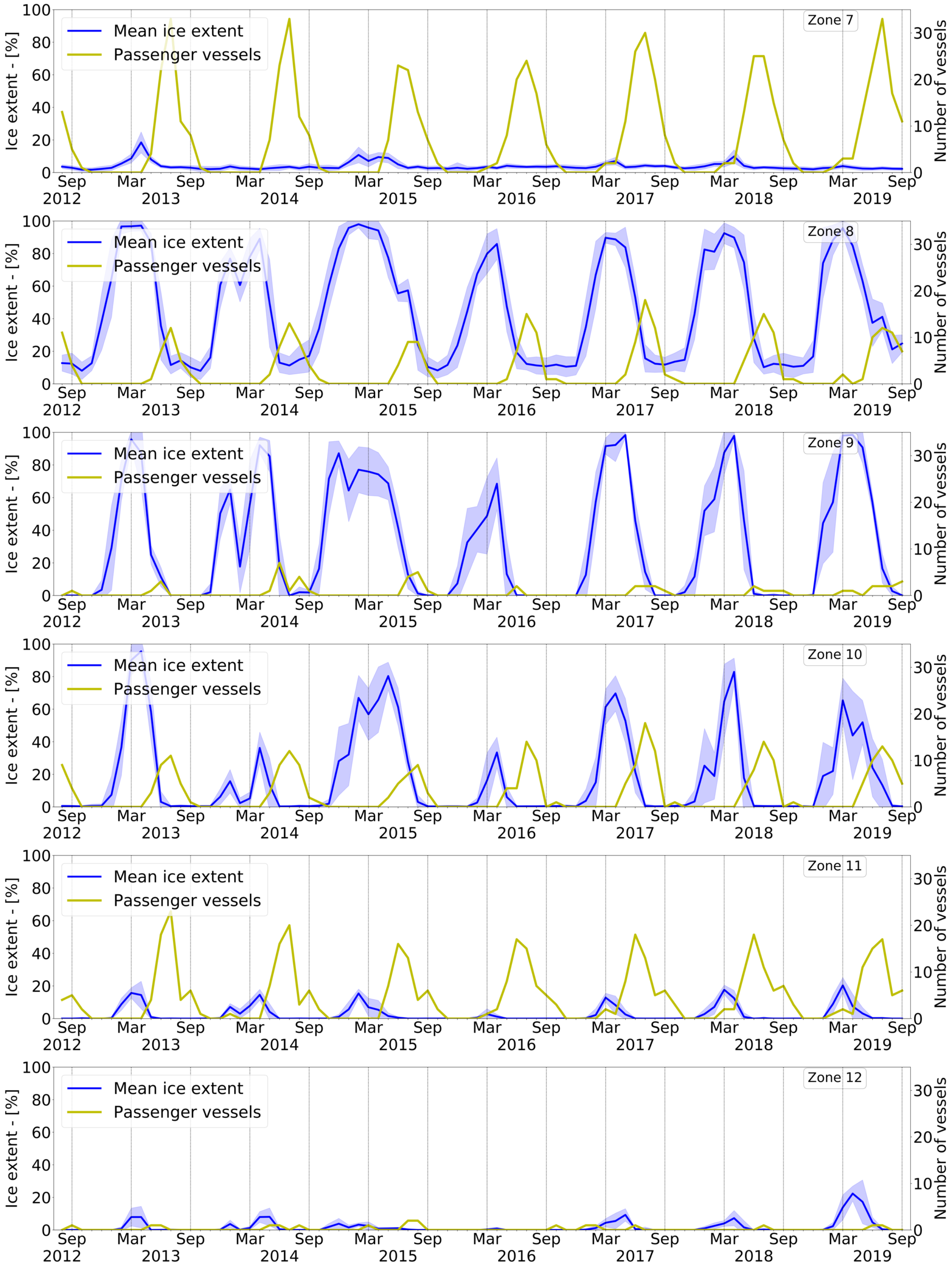

Figure S20: Time series of relative mean sea ice extent, with monthly standard deviation and number of passenger vessels in Zone7 to Zone 12 (see Figure S16).
